# PAD4 and Its Inhibitors in Cancer Progression and Prognosis

**DOI:** 10.3390/pharmaceutics14112414

**Published:** 2022-11-08

**Authors:** Di Zhu, Yu Lu, Yanming Wang, Yuji Wang

**Affiliations:** 1 Department of Medicinal Chemistry, College of Pharmaceutical Sciences of Capital Medical University, Beijing 100069, China; 2 Beijing Area Major Laboratory of Peptide and Small Molecular Drugs, Engineering Research Center of Endogenous Prophylactic of Ministry of Education of China, Beijing Laboratory of Biomedical Materials, Beijing 100069, China; 3 School of Life Sciences, Henan University, Kaifeng 475004, China

**Keywords:** PAD4, gene regulation, citrullination, NETosis, cancer, cancer-associated thrombosis, PAD4 inhibitor

## Abstract

The systemic spread of malignancies and the risk of cancer-associated thrombosis are major clinical challenges in cancer therapy worldwide. As an important post-translational modification enzyme, peptidyl arginine deiminase 4 (PAD4) could mediate the citrullination of protein in different components (including nucleus and cytoplasm, etc.) of a variety of cells (tumor cells, neutrophils, macrophages, etc.), thus participating in gene regulation, neutrophil extracellular trap (NET) and macrophage extracellular trap (MET). Thereby, PAD4 plays an important role in enhancing the growth of primary tumors and facilitating the distant metastasis of cancer cells. In addition, it is related to the formation of cancer-associated thrombosis. Therefore, the development of PAD4-specific inhibitors may be a promising strategy for treating cancer, and it may improve patient prognosis. In this review, we describe PAD4 involvement in gene regulation, protein citrullination, and NET formation. We also discuss its potential role in cancer and cancer-associated thrombosis, and we summarize the development and application of PAD4 inhibitors.

## 1. Introduction

The main challenge of cancer therapy relates to the systemic effects of malignant tumors, including metastasis and cancer-associated thrombosis (CAT), which are the leading causes of death in cancer patients [1,2,3]. For patients with focal solid tumors, surgical resection of the primary tumor is frequently the only chance for cure. However, the high recurrence rate of postoperative cancer and the risk of venous thromboembolism (VTE) pose tremendous challenges to clinical treatment, which often involves chemotherapy that is required after surgery to prevent disease recurrence [4,5,6]. However, certain chemotherapeutic agents used in cancer treatment (e.g., tamoxifen, cisplatin, and thalidomide) also increase the VTE risk and promote thrombosis through various mechanisms [7,8,9]. Therefore, the development of chemotherapy agents with dual antitumor and antithrombotic functions is of particular importance. In recent years, peptidyl arginine deiminase 4 (PAD4) has attracted considerable attention due to the key role it plays in cancer progression and CAT, which affects patient survival and prognosis and has become a potential target for cancer therapy.

**Figure 1:**
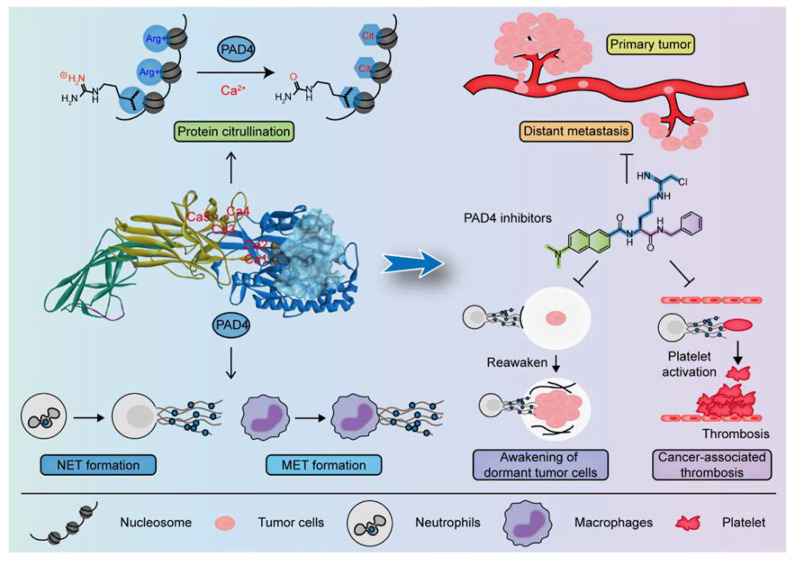
PAD4 and its inhibitors in cancer progression and prognosis. PAD4 promoted citrullination and NET formation in a calcium-dependent manner to induce tumor growth, metastasis, cancer cells awakening and cancer-associated thrombosis, which could be blocked by PAD4 inhibitors.

Protein-Arginine Deiminase (PADs, containing PAD1–4 and PAD6) is an essential post-translational modification (PTM) enzyme that converts protein arginine residues to noncoding citrulline residues in a calcium-dependent manner (Figure 1) [10]. Citrullination alters the structure, function and binding properties of target proteins, thereby enabling their participation in a variety of physiological and pathological processes [11,12]. Notably, PAD4 is the only PAD isoenzyme that carries a standardized nuclear localization sequence (NLS), which enables it to bind a diverse array of substrates in organisms. Among these proteins, histones are the most studied substrates. Histones can be citrullinated at different sites, altering their interactions with DNA and other nuclear proteins to activate or inhibit gene transcription. For example, histone H3 is citrullinated at R26 (H3R26) at estrogen receptor (ER) target promoters and enhances ER target gene expression by promoting local chromatin depolymerization [13]. In contrast, H3R17 citrullination antagonizes the transcriptional activation induced by methylation at H3R17, leading to the transcriptional repression of ER target genes [14]. PAD4 also participates in the suppression of p53 activity by modifying histones through citrullination, leading to the modulated expression of p53 target genes [15,16]. These studies support the idea that promoter-specific histone citrullination is involved in gene regulation. Another important study showing the effects of PAD4 on histone citrullination includes driving the formation of neutrophil extracellular traps (NETs), which comprise a host defense mechanism to prevent microbial invasion by bacteria, fungi, viruses and parasites [17,18,19,20,21]. PAD4-mediated protein citrullination is involved in the regulation of various physiological processes, and dysregulated citrullination has been shown to be associated with many autoimmune diseases, such as rheumatoid arthritis, multiple sclerosis, sepsis, Corona Virus Disease 2019 (COVID-19), cancer and thrombosis [22,23,24,25,26,27].

Pathological studies have reported PAD4 overexpression in a variety of malignancies, including breast carcinomas, lung carcinomas, hepatocellular carcinomas (HCCs), colorectal carcinomas, ovarian adenocarcinoma, uterine adenocarcinoma, bladder carcinomas, chondromas, and other metastatic carcinomas [28,29], suggesting PAD4 involvement in the tumorigenesis of multiple tissues. In the context of cancer, increasing evidence suggests that PAD4-mediated protein citrullination and Neutrophil/macrophage extracellular traps (NET/MET) formation play important roles in cancer progression not only by promoting the development of primary tumors but also by facilitating distant metastasis of cancer cells and CAT. Therefore, the development of potent and highly selective PAD4 inhibitors is of great importance. Considering these findings, in this review, we mainly summarize the classification and characterization of the PAD family; PAD4 involvement in gene regulation, protein citrullination and NET/MET formation; and the potential role played by PAD4 in cancer and CAT. Finally, the development and application of PAD4 inhibitors are discussed based on the structure and mechanism of PAD4.

## 2. Peptidyl Arginine Deiminase (PAD) and Citrullination

**Figure 2:**
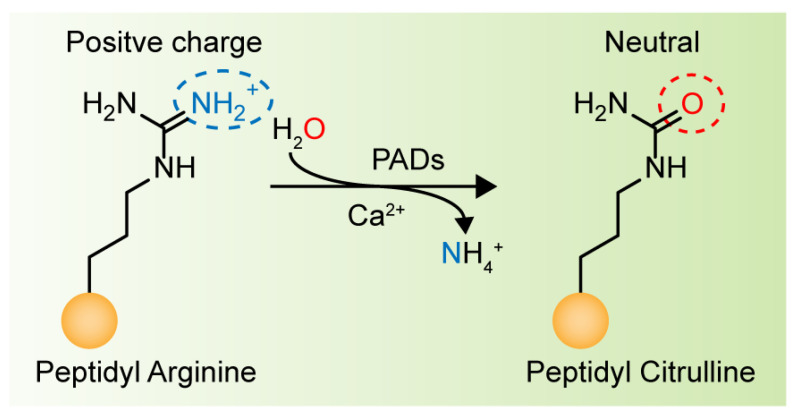
PAD-mediated citrullination: PADs catalyze the conversion of arginine to citrulline in protein with the participation of calcium ions.

Protein PTM greatly enhances the diversity of protein structures and functions, playing important roles in gene regulation and a variety of human diseases. PADs, constituting a family of calcium-dependent hydrolases, can irreversibly convert arginine residues into citrulline residues, known as citrullination, causing a marginal mass change (−0.02 Da compared to a charged guanidinium group, or +0.98 Da compared to neutral guanidine form, as indicated by mass spectrometry), leading to the loss of the arginine positive charge and an increase in protein hydrophobicity (Figure 2) [27,30,31]. Citrullination can significantly reduce the amino acid isoelectric point, from 11.41 for arginine to 5.91 for citrullination, resulting in changes in protein acidity and affecting the formation of hydrogen bonds and electrostatic interactions, thus changing the structures, functions and binding properties of modified proteins, and in certain cases, leading to protein denaturation [32,33,34]. Notably, free arginine cannot be citrullinated, and not all arginine residues in a protein are citrullinated with equal frequency. As reported, arginine residues adjacent to a proline or glutamic acid residue are negligibly citrullinated, whereas arginine residues near aspartic acid residues are frequently citrullinated [35,36]. In addition, arginine accessibility is determined by the secondary structure of a protein, which makes arginine residues in β-turns more readily citrullinated than those in α-helixes [35,36]. Although the human citrullinome is limited to approximately 200 types of proteins, most commonly vimentin, actin, collagen, fibronectin, filaggrin, keratin, tubulin, and various histones [37], PAD-mediated citrullination is considered to be a core factor in the transcriptional regulation of gene expression [38]. In particular, since citrullinated histones account for approximately 10% of all histone molecules in HL-60 granulocytes, the importance of this PTM in many nucleus-related processes has been highlighted [39]. However, the levels of citrullination are not highly correlated with the protein expression of PADs [37], suggesting that the PAD-mediated regulation of protein citrullination may be a complex and dynamic process. In other words, PADs are enzymes that need to be activated to cause citrullination. Hence, it is not the protein expression but rather the enzymatic activation and then downstream citrullination of target proteins. It will be further discussed in Section 8.

To date, five PAD isoenzymes have been found in humans and other mammals: PAD1–4 and PAD6 (PAD5 is a mouse homolog of PAD4), of which PAD6 shows no catalytic activity due to a mutation in the active site (Table 1) [40]. Although these enzymes are highly conserved and share more than 50% sequence similarity, they exhibit different tissue localization and substrate specificity, and therefore, they perform different functions. PAD1 and PAD3 are mainly distributed in the epidermis and hair follicles and are involved in epidermal differentiation and homeostasis [41]. Moreover, PAD1 distribution has been detected in the uterus [42,43]. Recently, researchers have also found that PAD1 inhibitors could also inhibit tumor growth and metastasis through the MEK1–ERK1/2–MMP2 pathway [44], and PAD3 inhibitors could kill PAD3-expressing HEK293T cells by rescue of thapsigargin-induced cell death [45]. PAD2 and PAD4 are widely expressed in human tissues. PAD2 is distributed in the brain, skeletal muscle, spleen and immune cells and is involved mainly in regulating nervous system function [46] and immune cell differentiation [47], while PAD4 is expressed in hematopoietic stem cells and immune cells (e.g., neutrophils, monocytes, macrophages and natural killer (NK) cells) [27,48,49]. Notably, PAD4 is the only PAD isoenzyme that carries a standardized nuclear localization sequence (NLS) [50], which enables it to target a range of nuclear proteins, such as histones (H1, H2A, H2B, H3 and H4), NPM1, ING4, P300/CBP, and lamin C. Furthermore, PAD4 is involved in apoptosis [36,51], gene regulation [52,53] and immune responses [54]. However, recent studies have shown that despite NLS deficiency, PAD2 undergoes calcium-mediated nuclear translocation [55], leading to the citrullination of histone H3R26, which mediates ER target gene activation [56]. PAD6, the most recently discovered PAD family member, is expressed mainly in the ovary, ovum and early embryo, where it is crucial for early embryonic development and female reproductive function [57,58]. Under most physiological conditions, PAD activity is strictly controlled and is activated only by high concentrations of calcium [36,59]. However, recent studies have shown that PAD can participate in gene regulation and other processes when the physiological calcium concentration is low, suggesting that other unknown PAD mechanisms are involved in gene regulation [36]. Although PADs are involved in the regulation of multiple physiological pathways, dysregulated citrullination can lead to disease progression or exacerbation. Increasing evidence suggests that the pathophysiological role played by PADs in several diseases makes them attractive therapeutic targets.

## 3. PAD Expression in Cancer and Associated Thrombosis

In recent years, the role played by PAD-mediated citrullination in cancer progression and thrombosis has been extensively studied. In the tumor microenvironment, factors such as nutrient deficiency, redox stress, or DNA damage can induce increased PAD activity and number of proteins that are citrullinated, influencing tumor progression and cancer treatment in a variety of ways.

PAD2 and PAD4 are the most widely expressed PAD family members. PAD4 in particular shows great potential as a cancer treatment target, which has been the focus of recent studies [80]. PAD2 has been reported to be highly expressed in tumor tissues and the blood of patients with one of several cancers, especially breast, liver, gastric and cervical carcinomas [81,82], and when overexpressed, PAD2 may suppress androgen activity in malignant tissues of castration-resistant prostate cancer (CRPC) patients [83]. However, PAD2 appears to be a tumor suppressor in colorectal cancer (CRC) patients [84]. In addition, the inhibition of PAD2 expression promoted the proliferation and migration of HCC cells [82]. These findings suggest that the effects of PAD2 on tumorigenesis are multifactorial and depend on tumor type.

In general, PAD4 is overexpressed in the tumor tissues and blood of patients with one of a variety of malignancies but exhibits low expression or is not expressed in benign tumors and normal tissues [28,29]. High PAD4 expression has been observed in approximately 40% of malignant lymphoma cells, suggesting that PAD4 may be involved in cancer development in all embryonic lineages [28,85]. Moreover, PAD4 levels were observed to be higher in metastases than in primary tumors, suggesting that PAD4 may promote a transition of benign tumors into invasive malignancies [86]. This evidence suggests that PAD4 is closely associated with tumor progression and may be a biomarker and potential target for cancer therapy. In addition, PAD4 in neutrophils drives NETosis and releases NETs triggered by stimuli in the tumor microenvironment, further promoting tumor growth and metastasis, inducing CAT, and thus resulting in poor prognoses [87,88]. Therefore, targeting PAD4 to prevent NET formation is a potential new direction for cancer treatment and a prognosis improvement strategy.

Recently, scholars have debated whether knocking out PADI4 gene expression in breast cancer cells can induce the epithelial–mesothelial transformation or enhance cell invasiveness [89] and whether restoring PAD4 expression in MCF-7/ADR cells induces apoptosis and reverses drug resistance [90]. These controversial findings suggest that the roles played by PAD4 in regulating tumor progression may be cell type- or environment-dependent. In the following sections, we focus on PAD4 and discuss the potential role it plays in cancer and CAT and the related mechanisms of its action.

## 4. The Structure of PAD4 and Its Mechanism of Action

Considering the potential role played by PAD4-mediated citrullination in the treatment of cancer and CAT, the development of efficient and specific PAD4 inhibitors is of great importance. Analyzing the structure of PAD4 can advance the understanding of the PAD4 mechanism of action and provide ideas for the design of PAD4 inhibitors.

### 4.1. PAD4 Structure

**Figure 3:**
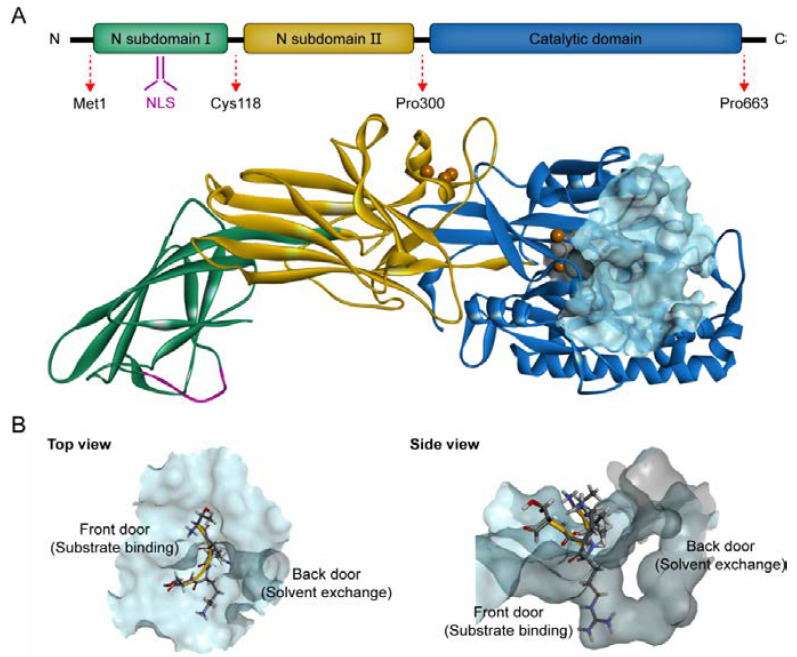
The three-dimensional structure of PAD4. (**A**) Calcium-bound PAD4 monomer structure (PDB: 1WD9). The NLS is shown in magenta, and the active pocket is shown in light blue. (**B**) The N-terminal tail of histone H3 occupies the PAD4 active pocket “front door” (PDB: 2DEW).

The PADI4 gene on human chromosome 1 encodes a 663-amino acid and a PAD4 monomer with a molecular weight of approximately 74 kDa [91]. The PAD4 monomer consists of two N-terminal immunoglobulin-like subdomains (subdomain I, residues Met1-Cys118, and subdomain II, residues Ala119-Pro300) and a C-terminal catalytic domain (residues Asn301-Pro663) (Figure 3A) [59]. Compared with the highly conserved C-terminal domain, the N-terminus of each PAD isozyme is significantly different, which affects PAD structural stability, substrate specificity and protein–protein interaction with proteases. The N-terminal domain of PAD4 includes an NLS (P56PAKKKST63), which is critical for PAD4 to interact with nuclear transporters to guide it through the nuclear pores, enabling it to target a series of nuclear proteins and initiate citrullination [40,91]. The PAD4 C-terminal domain includes its active site and forms an α/β propeller structure [59]. Under physiological conditions, PAD4 forms a head-to-tail homodimer with both active site cavities located on the same face [31,40]. This dimerization is stabilized by hydrophobic interactions and salt bridges established between adjacent monomers, and the ionic interaction between Arg8 and Asp547 and the hydrophobic interaction mediated by Tyr435 are the key factors for dimer formation [92]. The homodimerization of PAD4 plays an important role in cooperative calcium binding and its full enzymatic activity, while destruction of the dimer interface results in a 50–75% reduction in PAD4 catalytic activity [92]. These findings reveal a previously unknown inhibitory pathway of PAD4, which disrupts the formation of the PAD4 dimer, thereby preventing PAD4 activation.

The active site of PAD4 has a negatively charged U-shaped tunnel containing two doors: the “front door” is the true binding site for the side chain of the arginine substrate and derived inhibitors, and the “back door” forms a highly polar solvent channel that discharges the ammonia generated in its reactions and allows water molecules to enter subsequent hydrolytic reactions (Figure 3B) [31]. This substrate tunnel confers high substrate selectivity, allowing particular substrates to enter and bind to the active site, depending on the depth and shape of the tunnel. However, a drug molecule with high affinity for the backdoor does not appear to significantly block the active site or otherwise effectively inhibit enzymatic activity [93]. Therefore, designing substrates with appropriate chain length and targeting the “front door” is a major direction for the development of PAD4 inhibitors.

### 4.2. Calcium Dependence and The Catalytic Mechanism of PAD4

The crystal structure of PAD4 shows five Ca^2+^-binding sites. Ca1 and Ca2 are located in the C-terminal domain near the bottom of the active site cleft, and they are critical for calcium-induced conformational change. Ca3, Ca4 and Ca5 are located in the N-terminal domain and are necessary to stabilize the conformation of PAD4, which facilitates the appropriate positioning of Ca2, triggering the catalytic residue Cys645 to move ≥5 Å and to reach the correct position for nucleophilic attack [31,59]. PAD4 activity is strictly regulated by calcium, with activity increasing more than 10,000-fold after calcium binding [92]. However, the calcium concentration required for the maximum activity of PAD4 in vitro is between 100- and 1000-fold higher than that of activated cells [94]. Therefore, how this enzyme is activated in vivo is unclear, but it has been speculated that the calcium dependence of PAD4 is altered by specific PTMs or by binding proteins. For example, auto-Abs isolated from patients with rheumatoid arthritis increased the calcium sensitivity of PAD4 and activates it at physiological calcium concentrations [95]. Alternatively, PAD may temporarily localize to intracellular calcium channels that, when opened, provide instantaneous local calcium concentrations at a millimolar level sufficient to activate the enzyme [96,97].

**Figure 4:**
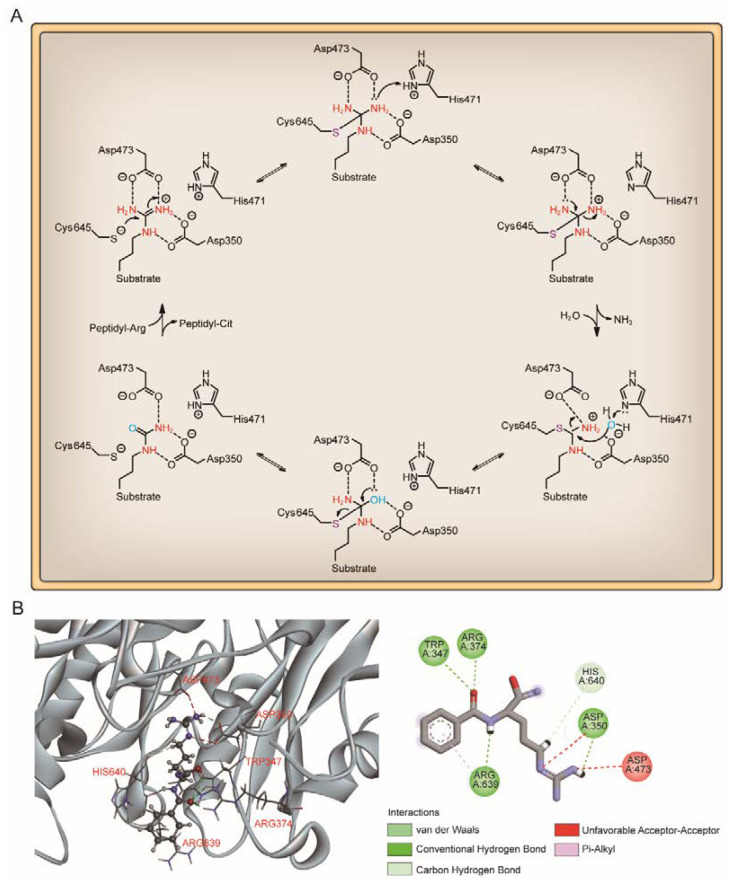
(**A**) Proposed catalytic mechanism of PAD4. (**B**) Interaction between PAD4 and the small-molecule substrate BAA (PDB: 1WDA). BAA is shown in a ball-and-stick model, and protein residues are shown in stick models.

Based on the crystal structure and biochemical studies, PAD4 enzyme catalysis has been suggested to follow an inverse protonation mechanism [59,98]. Four key residues in the PAD4 active site, Cys645, Asp350, Asp473 and His471, are essential for the conversion of arginine substrates into citrulline. Among these residues, Asp350 and Asp473 are located at the bottom of the tunnel and act as “anchor points”, which contribute to the correct positioning of the substrate side chain by forming hydrogen bonds with nitrogen atoms on the guanidinium group. The catalytic process is initiated by the Cys645 thiolate nucleophilic attack on the substrate guanidinium carbon to form a covalent tetrahedral intermediate. His471, located opposite the guanidinium group, is thought to promote catalysis by protonating tetrahedral intermediates, facilitating C-N bond rupture simultaneously or in a stepwise fashion through nucleophilic attack, leading to the release of ammonia and formation of a covalent S-alkylthiouronium intermediate; this intermediate is subsequently hydrolyzed by the attack of a His471-activated water molecule to form a second tetrahedral intermediate, which eventually collapses to eliminate the Cys645 thiolate and generate citrulline (Figure 4) [31,98]. Notably, the mutation of any of these four key residues leads to a significant reduction in enzymatic activity, suggesting that substrate binding, nucleophilic attack and acid–base catalysis are highly synergistic processes in citrullination [98].

## 5. PAD4-Mediated Protein Citrullination and Gene Regulation Components

### 5.1. Crosstalk between Components of PAD4-Mediated Histone Citrullination and Other Modifications

In eukaryotic cells, DNA with histone and nonhistone proteins make up the complicated structure of chromatin. Histone modifications, including methylation, acetylation and citrullination, play important roles in altering DNA accessibility and gene regulation. Among these PTMs, citrullination leads to a diminished positive charge of arginine-rich histones, which weakens the interaction of these proteins with the negatively charged DNA skeleton and leads to the opening of chromatin structure, promoting the binding of transcription factors to exposed DNA and thereby increasing the transcriptional expression of certain genes. Histones citrullinated by PAD4 can function with other modified histones to regulate gene transcription and induce tumor suppressor gene silencing, thus promoting tumorigenesis [99,100].

**Figure 5:**
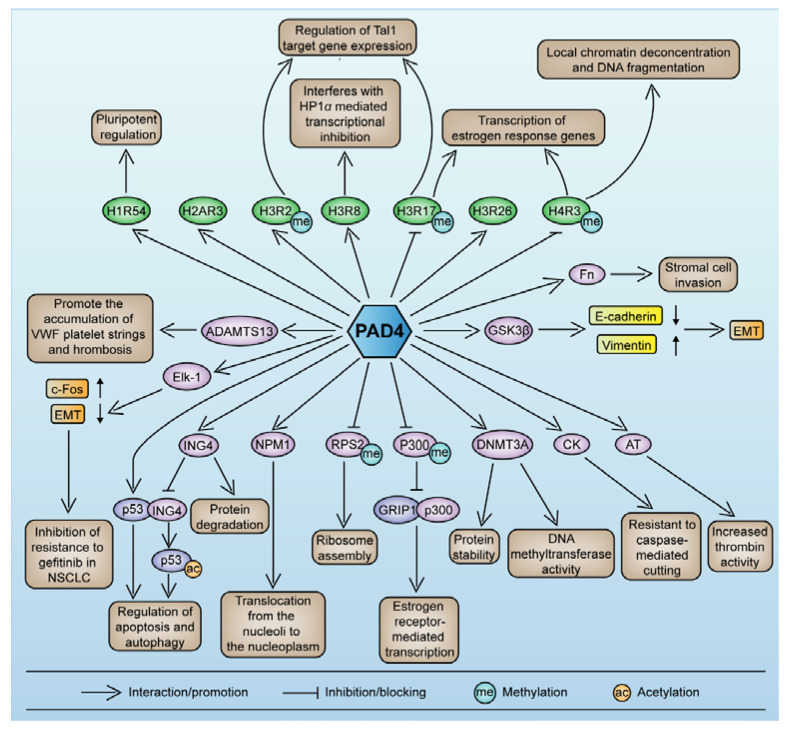
Schematic diagram showing the major substrates and regulatory mechanisms of PAD4 action. PAD4 citrullinates both histones (shown in green) and nonhistone proteins (shown in pink), regulating gene transcription alone or in combination with other PTMs. Me, methylation; Ac, acetylation.

During histone modification, enzymes involved in protein citrullination or methylation engage in competitive inhibition. For example, PAD4 antagonizes the methylation of histone arginine. It converts monomethylated (but not dimethylated) arginine into a citrullinated residue, causing the release of methylamine (effectively removing the methylation mark), or it directly citrullinates arginine to prevent a methyltransferase from adding a methylation mark on the arginine residue (Figure 5) [14,101,102]. Studies have shown that the citrullination of H3R17 by PAD4 on the ER-regulated pS2 promoter caused a significant decrease in estrogen-induced gene expression in MCF-7 breast cancer cells by interfering with methylation events mediated by CARM1 (also known as PRMT4) [14]. This study suggests that PAD4 may be a transcriptional regulator that antagonizes the transcriptional activation of arginine methylation, thereby regulating intricately controlled ER target gene expression. PAD4 can bind to the central hematopoietic transcription factor Tal1 and participate in other types of epigenetic regulation [103]. For example, it negates the inhibitory effect of the H3R2me2a mark deposited by PRMT6 on the *IL6ST* promoter, increasing IL6ST expression, and it similarly negates the effect of the H3R17me2a mark deposited by PRMT4 on the *CTCF* promoter to inhibit CTCF expression [103]. In addition, PAD4-mediated citrullinated marks may engage in a negative crosstalk relationship with methylated marks on adjacent lysine residues. For example, the citrullination of H3R8 by PAD4 significantly attenuated the H3K9me3-induced recruitment of heterochromatin protein 1α (HP1α) to ER-dependent promoters and thus interfered with HP1α-mediated transcriptional inhibition [104]. Furthermore, the citrullination of H3R8 in peripheral blood monocytes led to the expression of the downstream immune genes TNFα and IL-8 and ultimately to inappropriate T-lymphocyte activation and uncontrolled immune responses in multiple sclerosis [104].

In addition to methylation machinery, histone citrullination-related proteins cooperate with histone deacetylation proteins to repress gene transcription and contribute to carcinogenesis. PAD4 and histone deacetylase 1 (HDAC1) produce a transcriptional inhibitory chromatin environment and silence gene expression at the pS2 promoter in a cooperative and periodic manner [105]. Furthermore, PAD4 and histone deacetylase 2 (HDAC2) interact with p53 through distinct domains and simultaneously bind to the P21 promoter in response to DNA damage, thereby regulating gene expression [38]. A PADs inhibitor (Cl-Amidine) combined with an HDAC inhibitor (suberoylanilide hydroxamic acid, SAHA) showed an additive effect in inducing the expression of P21, GADD45, and PUMA, effectively inhibiting the growth of P53-dependent osteosarcoma (OS) cells [38]. This evidence indicates important crosstalk and dynamic associations between the enzymes involved in PADs-mediated citrullination and those of other PTMs to coregulate gene expression.

### 5.2. The Roles Played by PAD4 in Cell Differentiation and Apoptosis

PAD4 plays an important role in cell differentiation and apoptosis by regulating gene transcription. Pluripotent cells exhibit self-renewal potential and can differentiate into all somatic and germ cell lineages [106,107]. PAD4 is expressed in OCT4–GIP embryonic stem (ES) cell lines and induced pluripotent stem cells (iPS) [108]. The citrullination of the single arginine residue H1R54 within the DNA-binding site of H1 resulted in H1 displacement from chromatin and global chromatin depolymerization, which enhanced the expression of genes involved in stem cell development and maintenance [108]. A pharmacological inhibition of PAD4 activity led to a reduced percentage of pluripotent cells in early mouse embryos and significantly reduced cell reprogramming efficiency, suggesting that PAD4-mediated citrullination plays an important role in the regulation of pluripotency [108]. In addition, increased PAD4 expression has been observed during the granulocyte differentiation of HL-60 cells induced by all-trans-retinoic acid (ATRA) [109]. PAD4 regulates PU.1 expression in a SOX4-dependent manner and thus mediates the differentiation process [109]. Interestingly, treatments with the PADs inhibitors Cl-amidine and F-amidine induced the differentiation of HL-60 cells and HT-29 colon cancer cells, possibly by increasing p21 expression [110].

As a corepressor of p53 activity, PAD4 participates in inhibiting p53 transcriptional activity and regulating the expression of multiple p53 target genes, indicating the important role it plays in regulating cell cycle progression and apoptosis. Studies have shown that histone arginine methylation is related to the transcriptional activation of p53 target genes and that PAD4 is recruited by p53 to the promoter of these genes to reverse methylation, thereby inhibiting the expression of P21/CIP1/WAF1 and OKL38 and disrupting apoptosis and the normal cell cycle [15,16]. In a variety of PAD4-overexpressing cancer cells, treatment with PAD4 inhibitors or PADI4 short interfering RNA (siRNA) increased the expression of p53 target genes, leading to cell cycle arrest and apoptosis [15,16]. PAD4 has also been reported to inhibit the expression of the p53 target gene Sestrin2 (SESN2) and regulate autophagy through the mammalian target of rapamycin complex 1 (mTORC1) signaling pathway in U2OS (OS) cells [111]. In addition, PAD4 citrullinates the NLS region of ING4 and thus prevents ING4 binding p53, inhibiting ING4-promoted p53 acetylation and P21 expression [52]. This evidence suggests that PADI4 may function as an oncogene.

However, other studies suggest alternative outcomes of PAD4 action. PAD4 appears to be transactivated by p53 via an intronic p53-binding site, allowing p53 to regulate protein citrullination. After DNA damage, H4R3 and Lamin C can be citrullinated in a p53/PAD4-dependent manner, promoting local chromatin depolymerization and DNA fragmentation and mediating the apoptosis of the damaged cells [112]. In non-small-cell lung cancer (NSCLC) models, the level of H4Cit3 was negatively correlated with p53 protein expression and tumor size, and *PADI4*-knockout (KO) mice showed resistance to apoptotic stimuli and sustained decreased caspase-3 expression [112]. In addition, in the HL-60 and Jurkat hematopoietic cancer cell lines, increased PAD4 expression appeared to induce apoptosis by promoting cell cycle arrest and triggering mitochondria-mediated pathways [113]. These evidences suggest that the role played by PAD4 in apoptosis may be cell type specific.

### 5.3. PAD4-Mediated Nonhistone Protein Citrullination

PAD4-mediated histone and transcription factor citrullination is involved in tumor progression in various ways, including gene regulation, cell differentiation and apoptosis. PAD4 promotes tumorigenesis and CAT by mediating nonhistone protein citrullination both inside and outside cells.

Cytokeratin (CK) is a ubiquitous tumor-specific marker that regulates intracellular signaling and apoptosis by interacting with various related proteins. The colocalization of PAD4 and CK has been observed in breast, liver, lung, colon, rectal, esophageal, gastric, bladder, and other cancers [29]. An immunoprecipitation assay revealed the colocalization of PAD4 and CK, and PAD4 could citrullinate CK8, 18, and 19, making it resistant to caspase-mediated cleavage, thereby disrupting apoptosis and promoting tumor progression [29].

Antithrombin (AT) is a conformation-sensitive serine protease inhibitor that maintains the dynamic balance between coagulant and the anticoagulant systems by inhibiting plasma thrombin activity. Under pathological conditions, the citrullination of AT leads to structural changes and loss of anticoagulant function, resulting in increased thrombin activity [32]. Moreover, high levels of PAD4 and citrullinated AT (CitAT) have been detected in the blood of patients with various malignant tumors but not in that of patients with benign or no tumors, suggesting that PAD4-mediated citrullinated AT may promote malignant transformation of tumors [28]. Later studies have shown that dysfunctional thrombin activity promoted malignant cell proliferation, invasion, and metastasis as well as abnormal angiogenesis and fibrin deposition in tumor tissues, leading to CAT [114].

ADAMTS13, a disintegrin and metalloproteinase with thrombospondin type-1 motif-13, cleaves UL-VWF (von Willebrand factor) into smaller VWF fragments that show less hemostatic activity than the full-length protein, thereby inhibiting thrombosis [115,116]. Plasma PAD4 has been reported to citrullinate ADAMTS13 on specific arginine residues to inhibit endogenous ADAMTS13 activity markedly, prolonging VWF interaction with the vascular wall and increasing the risk of thrombosis [116]. These outcomes have been effectively demonstrated through the intravenous administration of r-huPAD4 in mice, and the inhibition of PAD4 activity by Cl-amidine blocked this effect [116]. Since PAD4 inhibition effectively prevented the accumulation of VWF platelet strings, the combination of a PAD4 inhibitor and ADAMTS13 may be a new treatment for thrombotic diseases.

Multiple citrulline substrates suggest PAD4 has the potential to be a therapeutic target for cancer and cancer-associated thrombotic diseases; however, the role played by PAD4 in regulating tumor progression, particularly the regulation of epithelial–mesenchymal transition (EMT), remains controversial. The EMT reduces epithelial cell polarity and adhesion properties, thereby enhancing the ability of cancer cells to metastasize and invade and the acquisition of a drug-resistant phenotype [117]. The descriptions of citrullinated substrates presented below suggest that PAD4 may be a complex tumor regulator.

Glycogen synthase kinase 3β (GSK3β) is a serine/threonine kinase that participates in a variety of signal transduction cascade reactions, inducing the EMT and regulating the expression of multiple cancer-related transcription factors [118,119]. In MCF-7 breast cancer cells, silencing the PADI4 gene led to a significant decrease in the nuclear GSK3β level, activation of TGF-β signaling, and induction of the EMT by decreasing E-cadherin and increasing vimentin expression, resulting in more aggressive tumor cells [89]. This effect depends on the PAD4-specific citrullination of GSK3β at N-terminal arginine residues, which promotes the nuclear localization and accumulation of GSK3β and reduces the expression of the EMT marker Smad4, thereby maintaining epithelial cell phenotypes [89]. Moreover, salvaging PAD4 expression in MCF-7/ADR cells has been shown to promote the nuclear accumulation of GSK3β and p53, which activates proapoptotic gene expression, thereby resensitizing cancer cells to adverse drug reaction (ADR)-induced apoptosis and reversing drug resistance [90]. These results suggest that PAD4 may suppress the tumor-promoting proliferation of breast cancer cells by regulating the nuclear level of GSK3β.

The erythroblast transformation-specific (ETS) domain-containing protein Elk-1, which is a member of the ETS family of transcription factors and is involved in cell differentiation, proliferation, apoptosis and tumorigenesis, is another complex PAD4 substrate [120]. In response to epidermal growth factor (EGF), PAD4 directly targets Elk-1 for citrullination, which promotes Elk-1 phosphorylation and histone H4 acetylation and in turn activates oncogene c-Fos expression [121]. Moreover, in another study, PAD4 overexpression inhibited the acquisition of a gefitinib resistance phenotype in NSCLC by inhibiting the Elk-1-mediated EMT, whereas the downregulation of PAD4 had the opposite effect [122]. These studies suggest that EMT regulation mediated by PAD4 may inhibit the acquisition of aggressive tumor and drug resistance phenotypes.

However, different results were obtained with the liver metastasis model of CRC. The study noted that higher levels of PAD4 and citrullinated proteins were detected in the extracellular matrix (ECM) of CRC liver metastases than in unaffected liver and primary CRC tissues [86]. Extracellular PAD4 may promote the transformation of epithelial cells into CRC cells through the citrullination of collagen type I and other ECM proteins, thereby enhancing cancer cell adhesiveness and inhibiting cancer cell mobility [86]. These outcomes were consistent with the observation that PAD4 overexpression inhibited the EMT of other cells. However, the inhibition of PAD4 activity, including citrullination, both enhanced mesenchymal cell marker expression and inhibited the growth of liver metastases [86]. Hence, the reversal of the EMT, that is, the mesenchymal–epithelial transition (MET), is believed to be necessary for initiating metastatic cell colony formation after transitioning [123,124].

Although multiple studies have shown that PAD4-mediated citrullination plays an important role in regulating EMT plasticity, the ultimate pathological outcomes are complex and may vary across different tumor types or different stages of tumor progression. This complexity is particularly manifested in the time window of targeted EMT therapy. Inhibition of the EMT to block tumor cell invasion seems to be an effective strategy to prevent early cancer metastasis; however, it may be counterproductive after tumor cells have emanated from the primary tumor because it may facilitate the reproliferation of circulating cancer cells and promote their colonization in distant organs [124].

In addition to the aforementioned substrates, PAD4 can citrullinate nonhistone proteins, such as p300/CBP [53], fibronectin [125,126], nucleophosmin (NPM1) [127], human ribosomal protein S2 (RPS2) [128] and DNMT3A [129], thereby partially affecting their activity or localization. The identification of PAD4-citrullinated substrates and the biological processes in which they are involved will help us understand the role played by PAD4 in the pathogenesis and progression of tumors and CAT and thus provides direction for cancer treatment and improved prognoses.

### 5.4. PAD4 Autocitrullination

PAD4 citrullinates a variety of protein substrates without showing clear specificity, except for a moderate affinity for RGG motif-containing substrates [130], making it a rather promiscuous enzyme. Interestingly, PAD4 can undergo autocitrullination, but whether autocitrullination influences its activity, such as through an autoregulatory process, is unclear [131,132]. A recent study showed that 85% of arginine residues in PAD4, including R372, R374 and R639 located near the active pocket were involved PAD4 self-modification [133], supporting the idea that PAD4 autocitrullination affects the interactions between PAD4 and its substrates but not the association between PAD4 activity and calcium dependence [132,133]. This supposition implies that the regulation of PAD4 activity may not be controlled through autocitrullination, and more studies are needed to analyze the role played by autocitrullination in vivo.

## 6. PAD4-Mediated NET Formation Is Involved in Cancer Progression

### 6.1. Key Role Played by PAD4 in NET Formation

As the first line of host defense in the innate immune response, neutrophils exert immune defense functions through phagocytosis, degranulation, the production of reactive oxygen species (ROS), and the release of cytokines, which recruit other immune cells [134,135,136]. In 2004, neutrophil extracellular traps (NETs) were first described as a novel mechanism in which neutrophils prevent the invasion of pathogens, representing the backup killing effect of phagocytic failure to capture escaping or large-sized microorganisms [17,137,138]. NETs are web-like structures composed of profoundly decondensed chromatin fibers bound with histones and decorated antibacterial proteins, including neutrophil elastase (NE), myeloperoxidase (MPO), matrix metalloproteinase 9 (MMP9), cathepsin G (CG) and peptidoglycan-binding proteins [17,139]. To date, three NET-induced mitochondrial DNA-releasing mechanisms have been identified, namely, suicidal NETosis, vital NETosis, and the vital NETosis that release mitochondrial DNA [140,141]. Most chemical irritants, such as phorbol myristate (PMA) [142], (auto) antibodies (Abs) [143,144], cholesterol crystals [145], and calcium ion carriers (A23187 and ionomycin) [146], have been shown to induce suicidal NETosis, which is a form of programmed cell death that differs from apoptosis and necroptosis [147]. Among these irritants, PMA is a powerful stimulant used to study NETosis in vitro. PMA activates protein kinase C (PKC) [146] and the Raf-MEK-ERK signaling pathway [148,149] to induce NADPH oxidase production of ROS, triggering MPO activation and NE release from granules. Subsequently, these two released enzymes translocate to the nucleus and cleave histones, causing chromatin decondensation and their eventual expulsion through the ruptured nuclear envelope and plasma membrane, resulting in neutrophil death [147,150]. However, ionophores can induce suicidal NETosis through a mechanism independent of NADPH activity [146], highlighting the complexity of NETosis pathways.

In addition, vital NETosis, also called “leukotoxic hypercitrullination” [151], happens following microbial-specific molecular patterns recognized by host pattern recognition receptors [150,152]. Vital NETosis is usually triggered by bacteria and their products in a ROS-independent manner mediated through Toll-like receptors (TLRs) [153,154,155] and integrin [156]-related mechanisms, causing neutrophil chromatin depolymerization and NET release in only a few minutes [156]. This rapid NETosis mode leads to the extracellular transport of NETs through vesicle exocytosis, preserving the plasma membrane integrity of neutrophils and maintaining the phagocytic and chemotactic functions of these cells, as well as their lifespan, which is not affected by DNA loss [157,158]. Thus, vital NETosis, through which mitochondrial DNA but not nuclear DNA is released, relies on ROS production but does not cause neutrophil death. This NETosis pathway is mediated through granulocyte-macrophage colony stimulating factor (GM-CSF) following lipopolysaccharide (LPS) or complement factor 5a (C5a) stimulation, resulting in NET release from 80% of neutrophils within 15 min [159]. Obviously, chromatin decondensation is an essential aspect of NETosis regardless of the mechanism through which the NETs are released [91,160]. Chromatin decondensation may be mediated by NE in granules, translocation to the nucleus and cleavage of histones in a ROS- and MPO-dependent manner, thereby causing their separation from the DNA [161,162]. Alternatively, PAD4 citrullinated histones may attenuate charge-based histone interactions with DNA, thereby promoting chromatin decondensation [12]. The relative importance of PAD4 to NETosis may be connected to the type of stimulus [151,163].

Nevertheless, the importance of PAD4 in NET formation has been demonstrated in several studies. For example, histone hypercitrullination has been detected in NETs formed after exposure to bacterial and inflammatory stimuli [164,165], whereas neutrophils from PAD4-KO mice did not show citrullinated histones, and they failed to respond to PMA, LPS, H_2_O_2_, bacterial or calcium ionophores to release NETs [166,167]. Notably, similar results have been obtained after the pharmacological inhibition of PAD4 activity [164,168]. PAD4 overexpression is not limited to granulocytes but can lead to NET-like structure formation in cells that normally do not form NETs, such as U2OS (OS) cells [169], suggesting that PAD4 is both necessary and sufficient for inducing chromatin depolymerization and DNA release. Although it has been suggested that PAD4 may not be necessary for suicidal NETosis induced by stimuli such as PMA [163,170], histones can be cleaved in an NE-dependent manner, thus exhibiting functional redundancy. Moreover, PAD4-mediated histone citrullination plays a key role in NETosis induced by various physiological stimuli, such as TNF-α, GM-CSF and fMLP [171]. These NET release processes are primarily ROS-independent, while the selective suppression of PAD4 in human neutrophils effectively prevents NET formation induced by these physiological stimuli [171]. Moreover, PAD4 activity is calcium-dependent; therefore, PAD4 has been shown to be activated by calcium ionophores to induce histone citrullination and NET formation [164,172]. In addition, neutrophils from PAD4-KO mice and differentiated human leukemia (dHL-60) neutrophil-like cells with disrupted PAD4 gene expression failed to respond to ionomycin-stimulated NET release [166,173]. This evidence suggests that PAD4 may be essential for chromatin depolymerization and NET formation in mouse and human neutrophils under physiological conditions.

The citrullination of connector-histone H1 and core histones H2A, H2B, H3 and H4 has been detected in NETs [164,174,175,176], with citrullinated histone H3 (H3cit) considered to be the most specific biomarker for circulating NETs [177]. However, the mechanism through which PAD4 is activated during NETosis in vivo remains unclear. For example, what concentration of calcium is required, and how does calcium production affect the mechanism of PAD4 activation? Whether other PAD4 substrates are involved in driving chromatin decondensation also remains to be clarified. Notably, PAD2 affects the NETosis process [178], but it does not appear to be necessary for NET formation; however, PAD4 is required [179,180]. NETs are regarded as a “double-edged sword”, playing a key role in the innate immune response but also participating in the development of multiple autoimmune diseases as well as noninfectious diseases such as cancer and thrombosis [181,182,183].

### 6.2. Effects of NETs on Cancer Progression and Prognosis

**Figure 6:**
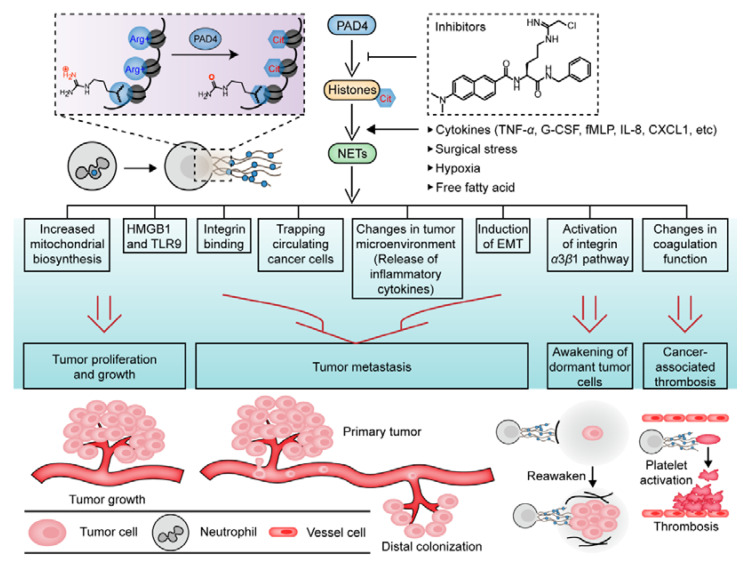
PAD4-mediated histone citrullination promotes NET formation, which in turn contributes to tumor progression and poor prognosis.

Neutrophils and NETs highly infiltrate tumor tissue, and neutrophilia in cancer patients is usually a predictor of poor prognosis [184]. The inflammatory/hypoxic environment of tumors and the secretion of various cytokines, such as TNF-α, IL-8, CXCL1, and granulocyte-CSF (G-CSF), recruit neutrophils and induce NETosis in tumors, thereby participating in multiple stages of cancer progression and promoting the malignant transformation of diseases (Figure 6) [185,186,187]. A recent study showed that NETs can directly alter the metabolic program of cancer cells by releasing NE and activating the TLR4–P38–PGC-1α pathway to increase mitochondrial biogenesis, thereby promoting cancer cell proliferation and tumor growth [188]. NETs also activate a series of TLR9-dependent signaling pathways, such as the MAP kinase pathway and NF-κB pathway, in cancer cells by releasing high mobility group box 1 (HMGB1), thus promoting cell proliferation, adhesion, migration, and invasion [189].

By promoting early adhesion events, NETs are associated with the progression of metastatic disease. As adhesion substrates in cancer cells, NETs promote the metastasis of cancer cells by binding to RGD-recognizing integrin, and this interaction is disrupted by preincubation with anti-integrin Abs [190,191]. Similar to its antibacterial functions, NETs can physically capture circulating cancer cells to facilitate metastasis and diffusion [192,193]. The NET network structures shield tumor cells from contact with cytotoxic T lymphocytes (CTLs) and NK cells, thereby helping cancer cells evade immune recognition [194]. In addition to direct interaction with cancer cells, NETs can cause changes in the tumor microenvironment, activating the TLR4/9–COX2 axis to trigger tumor-induced inflammatory responses and induce the release of inflammatory cytokines, such as TNF-α, IL-6, IL-8 and CXCL-10, thus promoting tumor growth and tumor cell metastasis and invasion (Figure 6) [189,195,196]. By upregulating the expression of various proinflammatory and EMT-related factors, NETs have been shown to promote the EMT, resulting in a more aggressive mesenchymal cell phenotype that promotes cancer metastasis [197,198,199]. NETs have also been associated with cancer recurrence and poor prognosis. For example, a recent study revealed that persistent inflammation induced by environmental toxins, such as tobacco smoke toxins, induces NET formation, which activates the integrin α3β1 pathway and FAK/ERK/MLCK/YAP signaling by releasing NE and MMP9 and triggering laminin cleavage and remodeling; this pathway awakens dormant cancer cells and promotes their proliferation [200]. This relationship between inflammation and toxins is similar to the close relationship between inflammatory stimulation, NETs and cancer recurrence in patients with long-term cancer dormancy.

Additionally, NETs alter coagulation in tumor patients and stimulate CAT, highlighting the importance of NETs in cancer progression and prognosis [87,201]. In fact, NETs regulate thrombosis through a variety of mechanisms. Cell-free DNA (cfDNA) is the main structural component of NETs, and it not only can activate coagulation factors FXI and FXII, triggering the endogenous coagulation pathway and increase thrombin production [202,203,204], but it can also provide a scaffold onto which platelets and red blood cells adhere; in addition, fibrin and platelet adhesion molecules, such as fibronectin and VWF, are deposited on these scaffolds, activating clotting cascades and promoting thrombosis [204,205,206]. Moreover, NETs can release extracellular histones, which induce epithelial and endothelial cell injury and lead to diffuse microvascular thrombosis [207,208]. These extracellular histones cause platelet aggregation and activation directly in a TLR2- and TLR4-dependent manner [209] or indirectly through fibrinogen [210]. These extracellular histones thus inhibit the activation of thrombomodulin-dependent protein C [211], thereby increasing thrombin production and promoting coagulation. Interestingly, the procoagulant capacity of a complete NET complex appears to be weaker than that of its individual DNA or histone components [204,212]. This finding may be explained by the fact that histones and DNA tightly interact in NETs, partially reducing the ability of each component to interact with the clotting system. In addition, NET-bound serine proteases such as NE and cathepsin G can enhance tissue factor- and FXII-dependent coagulation by inactivating tissue factor pathway inhibitor (TFPI) [213]. Notably, the interaction between NETs and platelets is not unidirectional. For example, released NETs bind to platelets and promote their activation, which in turn stimulates NET formation, establishing a positive feedback loop that promotes pathological thrombosis [214,215]. In fact, activated platelets promote NETosis during thrombosis through multiple pathways, including platelet–neutrophil interaction induced pathways mediated by p-selectin/p-selectin glycoprotein ligand-1 (PSGL-1) [216,217,218] and the release of damage-associated molecular patterns (DAMPs), such as HMGB1 [219,220,221] and platelet TLRs [153]. The interaction between NETs and platelets provides a new perspective for the clinical evaluation and treatment of thrombosis, and the effective intervention of CAT has important clinical significance.

Whereas NETs play a driving role in tumor growth, metastasis, and invasion and in cancer cell immune escape [189,194,206,222,223] and can awaken dormant cancer cells [200] and promote CAT [224,225], leading to the malignant transformation of tumors and poor prognosis, the degradation of NETs through DNase Ι or the inhibition of NETosis through PAD4 inhibitor treatment are important to the prevention and treatment of disease. However, DNase Ι cannot completely remove non-nucleotide NET components, such as histones and NE, despite its effectiveness in digesting extracellular DNA [226,227]. Nevertheless, the aforementioned NET-associated proteases can cause tissue damage and microthrombus formation even after the dissolution of NET structures. Therefore, the development of PAD4-specific inhibitors to prevent NET formation may be an effective treatment for cancer and CAT.

## 7. PAD4 in Cancers

PAD4 is overexpressed in a variety of malignant tumors, but it is expressed at a low level in normal and benign tissues [29,228]. The key role played by PAD4 in tumor progression and malignant transformation has been gradually explored through various experiments including those based on animal models and clinical studies. For patients with late-stage cancer, a correct diagnosis is essential to select the appropriate therapy and improve patient outcomes. A recent study proposed H3Cit as a novel diagnostic and prognostic blood biomarker for patients with advanced cancer [229]. A significantly increased average plasma H3cit concentration was detected in 60 advanced cancer patients with different malignancies compared with healthy individuals and patients that were seriously ill but without known cancer, and the level was not higher in the seriously ill patients without known cancer than in healthy people, supporting the idea of circulating H3Cit as a biomarker of advanced cancer and not a reflection of disease burden in general [229]. Notably, a high level of plasma H3Cit (>29.8 ng/mL, over 75%) was associated with a twofold increased risk of short-term mortality, implying a high correlation between elevated circulating H3Cit levels in cancer patients and their clinical load and prognostic outcomes [229]. In addition, plasma H3Cit levels were generally associated with higher cfDNA levels and neutrophil activation in the cancer patients, suggesting the involvement of NETs [192]. Moreover, the circulating H3Cit level was significantly correlated with high levels of IL-8 and IL-6 in the plasma of the cancer patients, which is important because these two cytokines have been shown to reflect the pathological progression and prognosis of cancer patients [230,231]. Therefore, circulating H3Cit alone or with other markers may be used for clinical diagnosis or prognosis for cancer in the clinic.

The detection of abnormal expression of PAD4 and its citrullinated substrates may provide additional opportunities for the early diagnosis and clinical intervention of cancers. However, the role played by PAD4 in certain cancers remains uncertain. Here, we summarize the potential role and underlying mechanisms of PAD4 in different tumor models (Table 2).

### 7.1. Gastric Carcinoma (GC)

GC is a common malignant tumor of the digestive system worldwide and a leading cause of cancer-related death. Compared with normal mucosal tissues, PAD4 is highly expressed in the malignant tissues and blood of GC patients and plays an important role in biological activities such as gastric cancer cell proliferation and invasion, cell cycle progression and apoptosis [28,29,233]. Notably, nucleotide variations in the tagged single-nucleotide polymorphisms (SNPs) rs882537 and rs1635566 in the PADI4 locus have been significantly correlated with gastric cancer, suggesting PADI4 is a susceptibility gene for gastric cancer [232]. Studies have shown that PAD4 can upregulate the expression of CXCR2, KRT14, TNF-α, and the matrix metalloproteinases MMP2 and MMP9, thereby activating the inflammatory response, angiogenesis, and cell proliferation, migration and invasion in tumor tissues, thereby promoting the development and progression of gastric cancer [232,233]. In addition, the inhibition of PAD4 expression by PADI4 siRNA resulted in arrest in the S phase and a reduction in the number of gastric cancer cells in the G2/M phase cell number reduction, specifically and significantly reducing the proliferative and invasive capacity of SGC-7901 and AGS cells [233]. In addition, PADI4 siRNA therapy together with 5-FU treatment enhanced the siRNA inhibitory effect on gastric cancer cell proliferation, suggesting that this combinatory regimen may help reduce the drug resistance of gastric cancer cells and improve therapeutic efficacy [233]. A recent study showed that PAD4 may promote the EMT and migration of GC cells by regulating IL-8 expression, which was inhibited by downregulating IL-8 expression [234]. This evidence suggests that PAD4 is involved in the pathogenesis of gastric cancer by regulating a variety of tumor-associated factors and is expected to be a potential therapeutic target and prognostic indicator for gastric cancer.

### 7.2. Ovarian Carcinoma (OC)

OC is the most frequent cause of mortality among gynecological malignancies. Due to difficulties in early detection, the prognosis of OC is poor. Compared with benign and normal ovarian tissues, PAD4 is overexpressed in most OC subtypes (e.g., serous cystadenocarcinoma, myxoid cystadenocarcinoma, clear cell carcinoma, and germ cell tumors) and is associated with the pathological grade of ovarian cancer [28,29,235]. PAD4 expression is regulated by estrogen. Increased PAD4 expression has been observed in SKOV-3 ovarian cancer cells incubated with 17β-estradiol (E2), and this increased expression showed a dose-dependent positive correlation with E2 concentrations in the ranging from 10–10 to 10–4 M [235]. Moreover, a significant association was apparent between PAD4 level and age of ovarian cancer patients. Because estrogen levels decline with age [258], PAD4 may be involved in tumorigenesis mediated through estrogen regulation, especially in postmenopausal women. In mechanism-based studies, silencing PADI4 by siRNA inhibited the proliferation of p53 wild-type A2780 cells and p53-null SKOV3 cells, suggesting that PAD4 is involved in the regulation of ovarian cancer cell proliferation mediated through a p53-independent signaling pathway [236]. Moreover, PADI4 siRNA treatment inhibited the invasion and migration but promoted the apoptosis of A2780 cells, while p53-null SKOV3 cells did not show these changes, suggesting that PAD4 may be involved in the invasion, migration and apoptosis of ovarian cancer cells in a p53-dependent manner [236]. In this study, a PCR array analysis revealed that 13 genes related to cellular behavior were differentially regulated, contributing to the understanding and elucidation of the detailed mechanisms by which PAD4 regulates the p53 signaling pathway and progression of ovarian cancer [236]. In addition, early ovarian cancers, especially those with high metastatic potential, induce the formation of NETs in the premetastatic omental niche, making these tissue favorable for ovarian cancer cell implantation [237]. PADI4 knockdown or PAD4 inhibitor treatment can effectively block NET formation and reduce omental implantation, providing new insight into improvements to the treatment of ovarian cancer and its common comorbidities [237].

### 7.3. Osteosarcoma (OS)

OS is the most common primary malignant bone tumor in children and adolescents, and it shows a high tendency for invasion and metastasis. Significantly upregulated PAD4 expression has been observed in OS tissues compared with that in adjacent tissues [28,29,239]. PAD4 promoted the proliferation of OS cells in vitro and in a mouse xenograft model primarily through the activation of the Wnt/β-catenin and MEK/ERK signaling pathways. Inhibition of PAD4 significantly downregulated p-MEK and P-ERK levels as well as the activation of β-catenin, effectively inhibiting the proliferation and colony formation of OS cells [239]. In addition, PAD4 reduced the expression of the epithelial marker E-cadherin and induced the EMT of OS cells, which are outcomes that were abrogated by PAD4 inhibitors [238]. Moreover, a positive correlation between PAD4 expression and the pulmonary metastasis of OS has been observed in clinical patient tissues [238], suggesting that PAD4 may be a promising target in OS treatment. Interestingly, PAD4 and HDAC2 have been shown to simultaneously bind the p21 promoter and thus corepress gene expression to inhibit tumor cell apoptosis [38]. Moreover, the combined application of the PAD4 inhibitor Cl-amidine or YW3-56 and the HDAC inhibitor SAHA showed an additive effect in inhibiting the proliferation of U2OS OS cells [38,111], providing a new strategy for OS therapy.

### 7.4. Nasopharyngeal Carcinoma (NPC)

NPC is a unique squamous cell carcinoma of the head and neck that is particularly common in East and Southeast Asia. Concurrent chemoradiotherapy is one of the major modalities of NPC treatment. Compared with that in normal tissues, PAD4 expression is upregulated in NPC tissues, differentiating PAD4 function from that of other PAD isoenzymes [240,241]. PAD4 overexpression has been shown to reverse the promoting effect of LINC00324 deletion on apoptosis and autophagy in mouse NPC cells and its inhibitory effect on tumor growth [242]. By interacting with miR-3164 and recruiting HuR protein in NPC cells, LINC00324 upregulates the expression of PAD4 to activate the PI3K/AKT pathway, thereby inhibiting autophagy and apoptosis in cancer cells and promoting the development of malignant tumors [242,259,260]. Silencing *PADI4* expression can inhibit the proliferation, increase the apoptosis rate and induce autophagy in NPC cells [242]. Analysis of apoptosis-related protein levels revealed that PAD4 overexpression downregulated the expression of the proapoptotic factors Bax, Bak, Bim and Bad; upregulated the antiapoptotic factors Bcl-2, Bcl-xl and Mcl-1; significantly reduced the proportion of cells in the G0/G1 phase, and promoted the cell cycle [241]. In addition, the upregulation of PAD4 expression inhibited the radiosensitivity of NPC cells and promoted their proliferation, migration and invasion, while PAD4 inhibitors, GSK484 and YW3-56 effectively reversed these effects and significantly inhibited tumor growth in mice [240,241], suggesting that the combination of PAD4 inhibitors and radiotherapy is a feasible treatment of NPC.

### 7.5. Hepatic Carcinoma

PAD4 is associated with the growth of primary hepatic carcinoma (PHC) and the liver metastasis of other cancers. Immunohistochemical results showed that PAD4 was significantly upregulated in the malignant tissues and blood of patients with PHC, including HCC and intrahepatic cholangiocarcinoma (ICC) [28,29,243]. HCC is the most common primary liver cancer and is characterized by cell invasion and metastasis, a poor prognosis, a high recurrence rate and high mortality. HCC onset is insidious and is usually diagnosed at an advanced stage [261]. In addition to surgical resection, chemotherapy is a common therapy for the treatment of advanced HCC, but the chemical resistance of HCC cells remains the main obstacle to the poor efficacy of chemotherapy. High PAD4 expression has been associated with postoperative chemotherapy resistance in HCC patients treated with transarterial chemoembolization (TACE), enhancing the chemical resistance of HCC cells in vitro and in vivo [243]. Notably, the overexpression of PAD4 in HCC cells induced autophagy, making these cancer cells resistant to a harsh microenvironment and chemotherapy, while autophagy inhibitors effectively restored the sensitivity of HCC cells to chemotherapy in vivo and in vitro [243], suggesting that PAD4 enhances HCC cell chemoresistance by inducing autophagy. In a recent study, 80 patients with hepatitis B virus (HBV)-related HCC presented with elevated H3Cit levels, which is associated with increased Beclin1 expression, suggesting a correlation between PAD4 and Beclin1 expression in the induction of HCC cell autophagy [244]. In addition, PAD4-mediated NET formation has been associated with HCC oncogenesis in mice with steatohepatitis, and the inhibition of NETs by DNase Ι or PADI4 KO has been shown to alter the pattern of liver inflammation and inhibited the development of HCC [245]. Increased NET levels have been observed in the serum of both nonalcoholic steatohepatitis (NASH) [245] and HCC patients [195], suggesting that PAD4 may be involved in the progression of hepatitis to liver cancer and contribute to the malignant transformation of liver disease into HCC. PAD4 can also trigger the metastatic potential of HCC cells by mediating NET formation and inducing enhanced cell death resistance and invasive ability, which have been shown to be inflammatory responses mediated via the internalization of NETs by HCC cells and activation of the TLR4/9–COX2 signaling pathway [195]. This study highlighted the role played by inflammatory factors in the metastatic potential induced by NETs and suggested that the combination of DNase Ι with the anti-inflammation drugs aspirin and HCQ can be used as a new strategy for HCC metastasis treatment [195]. In another study, positive feedback loops between neutrophils and cancer progression were enhanced by the inflammatory factor IL-8 and NET-associated cathepsin G, indicating that a simultaneous increase in CG and H3cit expression is necessary for HCC cell invasion [246]. Together, these studies support PAD4 as a potential therapeutic target to counter HCC progression and attenuate chemotherapeutic resistance.

Due to its abundant blood supply, the liver is one of the most common organs affected by tumor metastasis. PAD4-mediated citrullination of ECM proteins plays an important role in colorectal cancer (CRC) metastasis to the liver and metastatic growth [86]. Compared with those in normal liver, primary CRC, or adjacent colonic mucosa, liver metastases displayed higher levels of PAD4 expression, and a higher proportion of citrullinated protein was detected in the ECM of liver metastases [86]. Notably, PAD4 may be released into the extracellular environment via extracellular vesicles (EVs), and this released PAD4 may citrullinate collagen type I and other ECM proteins, which has been shown to promote the adhesion and inhibit the mobility of CRC cells, thereby inducing MET and liver metastasis growth [86]. The MET has been previously shown to be necessary for the successful growth of tumors at metastatic sites [123,124]. In experimental liver metastasis models, PADI4 KO or inhibition effectively inhibited the citrullination of ECM proteins, enhanced the expression of mesenchymal markers, and significantly inhibited liver metastasis growth [86]. Therefore, the inhibition of PAD4 and PAD4-mediated ECM protein citrullination may be potential targets for liver metastasis prevention and treatment.

For patients with metastatic CRC, resection of liver metastases, when feasible, remains an effective strategy to prolong survival [262]. However, liver recurrence after surgical resection is the main cause of treatment failure [263]. PAD4-mediated NET formation appears to be a potential contributor to metastatic cancer progression under surgical stress, which is supported by NET deposition in patients with Ewing’s sarcoma and primary CRC [223,247].

Surgical stress increases NET formation and contributes to metastatic tumor growth by promoting the establishment of metastases or the growth of existing micrometastases, while DNase Ι or PAD4 activity inhibition can effectively inhibit the promoting effect of NETs on tumor metastasis [189]. In vitro studies have shown that NETs induced changes in the inflammatory microenvironment and activated TLR9-dependent pathways in cancer cells by releasing HMGB1, thereby promoting cancer cell adhesion, proliferation, migration and invasion [189]. In addition, NETs can also release NE and activate the TLR4-p38-PGC-1α axis in cancer cells to increase mitochondrial biosynthesis, endowing cancer cells with survival advantages that leads to their migration and invasion and subsequent establishment of clinically difficult-to-detect micrometastatic disease, ultimately leading to tumor recurrence [188]. These findings suggest that NET degradation or PAD4 inhibition may be a potential therapeutic strategy for preventing tumor recurrence after surgery.

### 7.6. Lung Carcinoma

Lung carcinoma is the leading cause of cancer-related death worldwide, NSCLC accounting for approximately 80% of all diagnosed lung cancer cases [264]. Early diagnosis of lung cancer is the key to reducing mortality and improving prognosis; in fact, early diagnosis can increase the 5-year relative survival from 6% for patients with advanced metastatic lung cancer to 33% for regional stage and 60% for localized-stage lung cancer [265]. Compared with that in healthy tissues, PAD4 expression is higher in the malignant tissues and blood of lung cancer patients, indicating its potential involvement in tumorigenesis [28,29,248]. A circulating cfDNA analysis combined with the determination of pro-platelet basic protein (PPBP) and PAD4 expression can effectively distinguish healthy donors from NSCLC patients, which may facilitate early lung cancer diagnosis or screening detection [249]. In our recent study, the PAD4 inhibitor YW3-56 was combined with a Au nanodrug delivery system to target tumors and induce tumor cell apoptosis induced through chemical–photothermal therapy, significantly inhibiting tumor growth and lung metastasis in mice while showing good biosafety [250]. This outcome suggests the feasibility of combining the PAD4 inhibitor delivery system with photothermal therapy in cancer treatment. However, PAD4 expression was significantly downregulated in gefitinib-resistant NSCLC cells. PAD4 overexpression in NSCLC cells induced by pCMV-2a/2b-PAD4 transfection reversed resistance to gefitinib by inhibiting the Elk1-mediated EMT, while downregulated PAD4 expression led to the opposite effect [122]. These results seem to indicate that PAD4 functions as a tumor suppressor, suggesting a complex role played by PAD4 in NSCLC cell resistance.

### 7.7. Breast Carcinoma

Breast carcinoma is one of the most common malignancies in women worldwide, and PAD4 appears to play a complex role in breast carcinoma progression. The expression levels of PAD4 in the tumor tissues and blood of breast cancer patients are significantly higher than those of healthy controls [28,29,252]. Estrogen promotes PAD4 expression in MCF-7 breast cancer cells through both ER-mediated classical and nonclassical pathways [266], and PAD4 can be recruited to the promoter of estrogen-activated genes to inhibit transcriptional activation of ER target genes by antagonizing arginine methyltransferase [14,101]; in addition, it can synergistically activate the oncogene c-Fos [121] and Elk-1, thereby promoting cancer progression. In an ER+ MCF-7 breast cancer model, the stable knockdown of PAD4 led to a decreased nuclear GSK3β level, activation of TGF-β signaling and the EMT, thereby enhancing the invasive potential of breast cancer cells [89]. In addition, the downregulation of PAD4 expression has been observed in MCF-7/ADR cells, and restoring PAD4 expression promoted the nuclear accumulation of GSK3β and p53, which activated proapoptotic gene expression and downregulated MDR1 expression, restoring the sensitivity of cells to ADR and reversing drug resistance [90]. Consistent with the effects of PAD4 inhibition in ER+ breast cancer cells, endogenous PAD4 inhibited breast cancer progression by limiting the size of the CSC population in a variety of breast cancer models in vitro and in vivo, and this effect was associated with the epigenetic repression of the stemness master transcription factors NANOG and OCT4 [251]. These results suggest that PAD4 exerts tumor cell-autonomous suppressive effects and affects the progression of breast cancer, especially ER+ breast cancer.

In contrast, PAD4 has been shown to promote progression in triple-negative breast cancer (TNBC), a tumor lacking estrogen receptor, progesterone receptor (PR), and human epidermal growth factor receptor type 2 (HER2) expression. Compared with other breast cancer types, TNBC is more prone to local recurrence or metastasis to the lung and brain within 3 to 5 years of diagnosis and showed low sensitivity to radiotherapy and chemotherapy [267,268]. Therefore, finding suitable targets for TNBC therapy is of great importance. Notably, 4T1 mouse breast cancer cells with high PAD4 expression released cancer extracellular chromatin networks (CECNs) in vitro and in vivo, thereby promoting cancer cell metastasis [222]. *PADI4* KO in 4T1 cells effectively inhibited tumor growth in an allograft model and reduced lung metastasis [222]. On the other hand, the degradation of extracellular DNA, including that in CECNs, by DNase Ι effectively inhibited the pulmonary metastasis of *PADI4*-expresing 4T1 cells without affecting circulating tumor cells and reduced metastasis following intravascular perfusion [222]. In addition, the PAD4 inhibitor GSK484 has been shown to enhance the radiosensitivity of TNBC cells in vitro and in vivo, leading to enhanced antitumor and antimetastatic effects by facilitating apoptosis and radiation-induced DNA damage in cells exposed to irradiation (IR) [253]. These outcomes suggest that PAD4 inhibitors can be used as radiosensitizers to improve the efficiency of radiotherapy in TNBC treatment. A recent study showed that PAD4 was recruited by hypoxia-inducible factors (HIFs) to the hypoxia response elements (HREs) of target gene under hypoxia, stabilizing the binding of HIFs to HREs by mediating local histone citrullination and leading to increased transcription and vascular endothelial growth factor expression. The interaction between PAD4 and HIF-1α has been shown to promote breast cancer angiogenesis and tumor growth in animal models and breast cancer patients [254], suggesting that PAD4 inhibition may be a novel treatment strategy for TNBC.

In addition, PAD4 may be involved in the activation and proliferation of breast cancer cells by mediating NET formation under inflammatory conditions. During lung inflammation induced by tobacco smoke or LPS, neutrophils released NETs and induce proteolytic laminin remodeling, activating the integrin α3β1 pathway and FAK/ERK/MLCK/YAP signaling, which awakened dormant cancer cells and led to lung cancer recurrence in mice. Fortunately, both DNase I and GSK484 treatment can prevent or attenuate the awakening and proliferation of dormant D2.0R and MCF-7 cancer cells [200]. Similar experimental results have been obtained in ozone-induced pulmonary metastatic cell models of breast cancer [255]. These findings suggest the potential effect of PAD4 inhibitors in preventing cancer recurrence and potential benefits of systemic PAD4 therapy administered at different pathological stages.

### 7.8. Leukemia

PAD4 was initially detected in HL-60 human myeloid leukemia cells and in granulocytes and monocytes induced by ATRA and 1α, 25-(OH)2D3 and is known to be involved in the differentiation and development of these cells [256]. Acute promyelocytic leukemia (APL), a hematological malignancy, is characterized by impaired cell differentiation and abnormal cell self-renewal, and treatment with ATRA promoted the stable increase and nuclear translocation of PAD4, regulating PU.1 expression in a SOX4-dependent manner and promoting the differentiation of leukemia cells [109]. This regulatory mechanism has been observed in both PML-RARα-negative (HL-60) and PML-RARα-positive (NB4) cells, suggesting that the PAD4/SOX4/PU.1 functional axis plays an important role in abnormal APL differentiation [109]. PAD4 has also been shown to regulate the proliferation of multipotent progenitor cells (MPPs) in bone marrow by binding LEF1 and HDAC1 to control the expression of the *c-myc* oncogene [257]. Lineage− Sca-1+ c-Kit+ (LSK) cells in mouse bone marrow, especially MPP cells, proliferated abnormally in an autonomous manner in *PADI4*-KO mice compared to WT mice [257]. PAD4 stimulated p21 and Bax transcription in a p53-dependent manner and induced apoptosis and cycle arrest, thereby inhibiting HL-60 and Jurkat (human acute T-cell leukemia) cell proliferation [113,269]. This evidence suggests that PAD4 is a tumor suppressor in the pathogenesis of leukemia.

## 8. PAD4 and Cancer-Associated Thrombosis (CAT)

Cancer patients are commonly in a hypercoagulable state and typically possess a higher risk of CAT, including arterial and venous thromboembolism. Advanced metastatic cancer has been associated with a higher risk of VTE (venous thromboembolism) than early local tumors, severely affecting patient survival and prognosis [270,271]. Cancer cells release proinflammatory cytokines, particularly G-CSF, to induce neutrophil proliferation and the activation and release of NETs, thereby promoting a prethrombotic state and CAT [272,273]. In addition, extracellular vesicles have been shown to induce neutrophils in G-CSF-treated mice to form NETs, and CSF combined with NETs synergistically promoted venous thrombosis in tumor-free mice [274,275]. Interestingly, NETs have been detected in both venous and arterial thrombosis [276,277], where they not only act as physical scaffolds for the adhesion and aggregation of platelets and red blood cells but also bind plasma proteins such as fibrinogen, VWF and fibronectin to activate coagulation cascades and further promote thrombosis [204,278]. Multiple components in NETs, especially cfDNA and histones, can trigger platelet activation and increase thrombin production, which exerts a profound impact on thrombosis [279,280]. Levels of these NET biomarkers have been associated with the severity of thrombotic disease, including thrombotic microangiopathy and deep vein thrombosis [281,282,283]. In addition, elevated levels of plasma cfDNA and circulating NETs have been observed in many different types of malignancies in close association with spontaneous thrombosis [274]. Compared with that in tumor-free mice, the degree of arteriovenous thrombosis was increased in tumor-bearing mice, while the NET biomarker H3Cit was directly detected within thrombi [284]. Moreover, NETs have been observed in the thrombi of cancer patients [285,286]. Notably, cancers can induce a higher rate of systemic NET formation than local NET formation, which is primarily observed in sterile and nonmalignant thrombotic diseases, suggesting that circulating NET markers can be used to predict CAT. Interestingly, a recent clinical study showed that the plasma H3Cit level was related to the occurrence of VTE in patients with lung and pancreatic cancer but not with other cancer types, such as breast or colon cancer [177]. This outcome means that circulating NET markers can be used as markers for VTE risk in patients with specific types of cancer.

Given the critical role played by PAD4 in NET formation, an increasing number of studies have focused on PAD4 involvement in thrombosis. These studies have revealed that the ability of PAD4-KO mice to generate thrombi after undergoing induced inferior vena cava stenosis was much lower than that of wt mice, and the H3Cit level was negligible in the generated thrombi [167]. Knockdown or inhibition of PAD4 activity significantly reduced the aggregation of neutrophils and platelets in the area of vascular injury, thereby preventing venous thrombosis in heparin-induced thrombocytopenia (HIT) [287]. PAD4 is a key factor in pathological thrombosis not only because it mediates NET formation but also because it citrullinates serine protease inhibitors, such as antithrombin, fibrinogen and VWF lyase (ADAMTS13), causing coagulation dysfunction and promoting thrombosis [116,288]. High levels of CitAT have been detected in the blood of patients with one of various cancers, and citrullinated CitAT induced structural changes and a loss of anticoagulant function, thereby enhancing thrombosis [28,32]. In addition, circulating PAD4 has been shown to citrullinate ADAMTS13, thereby inhibiting its ability to cleave UL-VWF, leading to the accumulation of VWF platelet strings on vascular walls and increasing the risk of thrombosis [116,289]. On the basis of accumulated ADAMTS13 in NETs, it is suggested that the PAD4-mediated citrullination of hemostatic proteins may enhance the ability of NETs to induce thrombosis. In conclusion, targeting PAD4 inhibition may be an effective strategy to prevent and combat CAT.

## 9. PAD4 Inhibitors

### 9.1. Pan-PAD and PAD4 Inhibitors

Based on the crystal structure and catalytic mechanism of PAD4, we describe several potential PAD inhibitor designs: compounds that disrupt the structure of the PAD4 dimer, compounds that occupy the “front door” of the U-shaped tunnel, and guanidine-containing compounds and their analogs (e.g., haloacetamidine compounds) that interact with catalytic residues in the active site. However, the highly conserved structure of all PAD isozyme active sites makes designing and developing selective PAD4 inhibitors a challenge. Here, we present several PAD4-specific and pan-PAD inhibitors to advance the understanding of the potential value of PAD inhibitors in cancer treatment (Table 3).

Initial studies focused mainly on the identification of several reversible PAD inhibitors, including Taxol, benzoyl-Nω, Nω-dimethylarginine (Bz-ADMA), streptomycin, minocycline, tetracycline, aureomycin, ruthenium red and sanguinarine [27,31,40,290,292]. However, due to their poor selectivity and catalytic activity that was realized only at high micromolar to millimole concentrations, their application prospects as PAD inhibitors were not ideal. Fortunately, GSK121 was identified as a lead compound for PAD4 inhibitor development from a DNA-encoded small-molecule library [168]. On this basis, lead compound optimization resulted in the more efficient and reversible PAD4-specific inhibitors GSK199 and GSK484, which significantly inhibited PAD4-mediated protein citrullination and NET formation with human and mouse neutrophils [40,168]. Notably, both compounds require calcium to exert their inhibitory effects and preferentially bind to calcium-free PAD4. In the absence of calcium, GSK199 and GSK484 inhibited PAD4 with half-maximal inhibitory concentration (IC50) values of 200 and 50 nM, respectively, while their potencies were reduced more than 5-fold in the presence of 2 mM calcium [168]. In addition, the crystal structure of the PAD4/GSK199 complex revealed that the Phe634 residue, which is unique to PAD4, is packed tightly with the benzimidazole moiety of the compound, which may lead to a strong affinity for PAD4 (>35-fold) [168]. Recently, several novel reversible PAD4 inhibitors have been described, such as furan-containing peptide-based inhibitors (Inh-Dap is the most promising) [293] and four potential PAD4 inhibitors (compound 4: SC97362 is the most promising) [294], which are expected to provide a design template for reversible PAD4 inhibitor development.

Reversible PAD4 inhibitors have been the focus of researchers’ efforts because they are diverse and complex chemical structures. However, they are still far from becoming mechanism-based PAD4-inhibiting drugs. In contrast, irreversible PAD inhibitors typically act on calcium-bound PAD and possess a more specific inhibition mechanism involving the covalent modification of the cysteine-catalyzed active site residues. NSC95397 and streptonigrin were initially identified as irreversible PAD inhibitors by a high-throughput screening platform based on ABPP [295,296]. Both inhibitors contain an α, β-unsaturated carbonyl functionality, which can covalently modify the active site cystine [40]. Interestingly, streptonigrin shows excellent potency and PAD4 selectivity, which may benefit from its substituted pyridine groups and benzene rings [302]. In addition, on the basis of the PAD4 small-molecule substrate benzoyl-l-argininamide (BAA), Thompson et al. replaced the original guanidine group with a haloacetamidine warhead and developed the first generation of highly effective and irreversible pan-PAD inhibitors Cl-amidine and F-amidine [297,303]; both of these compounds covalently modify the active site cystine in a time- and concentration-dependent manner and irreversibly inactivate PAD4 and other PAD isoenzymes in a calcium-bound state [297,303]. Between these two inhibitors, Cl-amidine is the most widely used compound and serves as a benchmark to evaluate the efficacy of other novel PAD inhibitors; it effectively inhibits histone citrullination and NET formation [304] and has ameliorated disease severity in several animal models [110,305,306,307]. Subsequently, the second generation PAD inhibitors o-Cl-amidine and o-F-amidine were generated by introducing a carboxylic acid into the ortho-position of the phenyl group, which effectively improved the inhibitory effect and selectivity for the PAD enzymes and enhanced the inhibition of H3 citrullination in HL-60 cells [298]. Alternatively, based on amide bond isosteres, BB-Cl-amidine and BB-F-amidine were obtained by introducing an N-terminal biphenyl group and a C-terminal benzimidazole group, which not only increased the hydrophobicity of the compounds but also improved their potency, bioavailability and stability in cells [40,299]. BB-Cl-amidine showed enzymatic inhibition and selectivity similar to those of Cl-amidine but had a longer half-life in vivo and 20-fold increased cytotoxicity in U2OS cells [305]. Moreover, Thompson et al. identified two peptide-based PAD inhibitors carrying a haloacetamidine warhead in a small peptide library: Thr-Asp-F-amidine (TDFA) and Thr-Asp-Cl-amidine (TDCA), TDFA being highly selective for PAD4 and showing significant efficacy in inhibiting H3 citrullination in HL-60 cells and with TDCA showing equivalent affinity for PAD1 and PAD4 [40,300].

However, most peptide-based PAD inhibitors exhibit high hydrophilicity, which contributes to their poor cell permeability and metabolic stability and, therefore, poor bioavailability and weak cell-killing ability. To enhance PAD inhibitor cell permeability, our research group modified the Cl-amidine scaffold with a Cα-amide-methylbenzene and an Nα-amide-dimethylnaphthylamine moiety and developed YW3-56 as a pan-PAD inhibitor with enhanced activity. YW3-56 showed an improved in vitro inhibitory effect on PAD4 enzymatic activity (IC50 is 1–5 μM) and a significantly enhanced cytotoxic effect on U2OS cells (IC50 is ≈2.5 μM; 60-fold that of Cl-amidine) [111]. Mechanistically, this novel pan-PAD inhibitor suppressed H3 citrullination and activated p53 target gene expression, including SESN2, thereby inhibiting the downstream mTORC1 signaling pathway and interfering with autophagy to inhibit the proliferation of cancer cells [111,308]. YW3-56 has been shown to be a potent inhibitor of tumor growth and metastasis in a variety of preclinical tumor models and has been shown to significantly improve the therapeutic effect when combined with radiotherapy, photothermal therapy, or chemotherapy (e.g., the HDAC inhibitor SAHA) [111,241,250]. Recently, we developed a novel PAD4 inhibitor, ZD-E-1M, which was based on YW3-56 with the Nα-amide-dimethylnaphthylamine moiety replaced with nitro-benzofurazan, which inhibited PAD4 enzyme activity when applied in the low-micromolar range in vitro and showed potent antitumor and antimetastatic activities in a 4T1 breast cancer model by inhibiting H3 citrullination and NET formation [301]. Furthermore, we observed that ZD-E-1M self-assembled in a pH-responsive manner and participated in the regulation of the tumor immune microenvironment, increasing the proportion of dendritic cells (DCs) and CD4+ T cells but reducing the proportion of myeloid suppressor cells (MDSCs) and LAG3 abundance on the surface of various immune cells [301]. Moreover, ZD-E-1M may be a potential CTLA4 inhibitor, showing enhanced antitumor activity in vivo when combined with an anti-PD1 Ab (αPD1) [301]. However, whether PAD4 regulates the tumor immune microenvironment and the mechanism of its involvement are unclear and will be the focus of our future work.

Although several pan-PAD inhibitors have been shown to exhibit excellent anticancer potential in a variety of preclinical models, these candidates may function through multiple mechanisms and affect different biological pathways. Therefore, the development of PAD4 inhibitors with high efficiency, high selectivity and improved bioavailability remains critical to elucidate the role played by PAD4 under physiological conditions and during disease progression to generate a potential therapeutic option for PAD4-dependent diseases, especially cancer and associated thrombosis [309].

### 9.2. Mechanism of Haloacetamidine PAD Inhibitors

**Figure 7:**
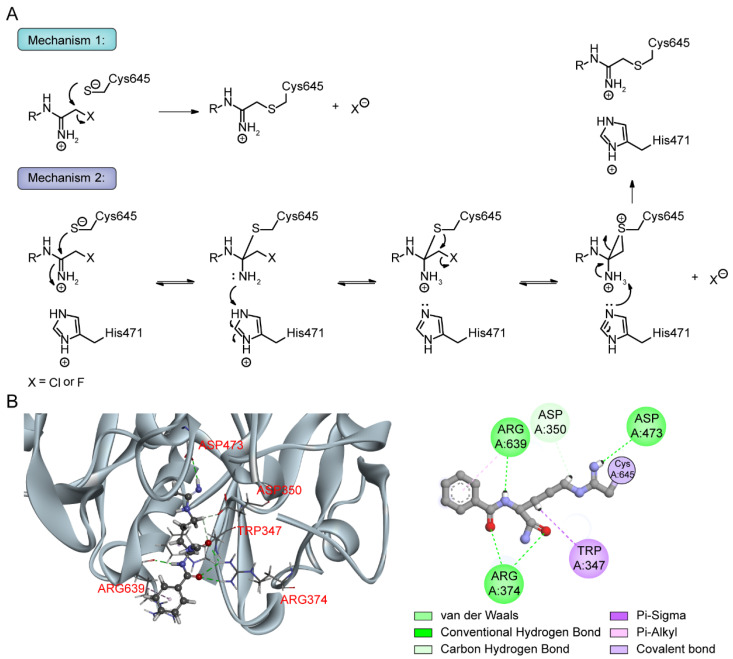
(**A**) Potential inactivation mechanism of haloacetamidine PAD inhibitors. (**B**) Interactions between PAD4 and the PAD inhibitor F-amidine (PDB: 2DW5). F-amidine is shown in a ball and stick model, and protein residues are shown in stick models.

To date, a substantial number of studies have focused on the design and development of haloacetamidine PAD inhibitors, which are well-established mechanism-based PAD inactivators that covalently modify catalytic cysteine residues in the active site in a calcium-dependent manner [310,311]. On this basis, Thompson et al. proposed two mechanisms to explain how haloacetamidine inhibitors inactivate the PAD4 enzyme (Figure 7) [297,312].

In the first mechanism, inactivation is achieved through an SN2 mechanism in which the halide portion of the inhibitor warhead directly displaces the Cys645 catalytic residue in the active site. This displacement inhibits the formation of the covalent tetrahedral intermediate needed for PAD4 to convert the guanidine of arginine into the ureido of citrulline. Alternatively, inactivation is achieved via a multistep mechanism initiated with the formation of the tetrahedral intermediate by the nucleophilic attack of the Cys645 thiolate on the amidinium carbon of the inhibitor warhead. Subsequently, His471 acts as a proton donor to promote halogen removal and form a ternary sulfur ring, which eventually collapses and rearranges to form a stable sulfur ether product, thus firmly holding Cys645 and inhibiting the catalytic function of the enzyme [31,297,313]. Although both mechanisms are plausible in principle, the observed bell-shaped pH inactivation rate curve of the enzyme strongly supports the second mechanism because it explains the proton donation step, the low leaving group potential of fluoride and the reduced ability for enzyme inactivation. Drug design based on this mechanism may contribute to the development of effective PAD4 inhibitors.

## 10. Conclusions

In recent years, increasing evidence has shown that PAD4 plays a key role in cancer progression and patient prognosis by participating in gene regulation, protein citrullination and NET formation. In this review, we summarize these findings and focus on the potential role played by PAD4 and its mechanisms of action in cancer progression and CAT. Finally, several potential inhibitors have been proposed based on the structure and catalytic mechanism of PAD4, and a variety of these PAD inhibitors are thus also reviewed. However, the mechanism through which PAD4 is activated in vivo and its potential role in cancer progression and related mechanisms remain to be explored. In addition, the design and development of PAD4 inhibitors that combine isotypic specificity with high potency will also be the focus of future efforts. In conclusion, we hope that the summary presented in this review will help elucidate the potential role played by PAD4 in cancer and associated diseases and provide ideas for the development of PAD4 inhibitors with antitumor and antithrombotic dual functions.

## Figures and Tables

**Table 1:** Distribution, substrates, and biological functions of PADs and related diseases.

PAD Isozyme	Expression Profile	Substrates	Physiological Roles	Pathological Roles
PAD1	Immune cells, keratinocytes, hair follicles, epidermis, uterus	Keratin, filaggrin, S100A3, MEK1-ERK1/2-MMP2 signaling enzymes	Skin differentiation, terminal keratinocyte differentiation, the epithelial-mesenchymal transition (EMT)	Psoriasis [60], breast carcinomas [44]
PAD2	Brain, skeletal muscle, spinal cord, glial cells, colon, pancreas, mammary gland, salivary gland, secretory gland, thymus, spleen, immune cells, bone marrow, yolk sac, epidermis, eye	MBP, GFAP, S100A3, CXCL10, CXCL11, Vimentin, *β*/*γ*-actin, histones (H3 and H4)	Oligodendrocyte differentiation, myelination, central nervous system (CNS) plasticity, transcription regulation, innate immunity, female reproduction	Multiple sclerosis [61,62], Alzheimer’s disease [63,64], rheumatoid arthritis [65,66], prion disease [67,68] and cancer
PAD3	Epidermis, hair follicles	Filaggrin, vimentin, trichohyalin, S100A3	Skin differentiation, hair follicle formation, terminal keratinocyte differentiation	Loss of barrier function and epidermal homeostasis [69], uncombable hair syndrome [70], glioblastoma multiforme [71]
PAD4	Immune cells (neutrophils, monocytes, and macrophages), brain, uterus, joints, bone marrow, breast, epithelial cells, cancerous tissues	Histone H1 (R54Cit), H2A (R3Cit), H3 (R2Cit, R8Cit, R17Cit, R26Cit) and H4 (R3Cit, R23Cit), Lamin C, ING4, cytokeratin, antithrombin, ADAMTS13, GSK3*β*, Elk-1, collagen type I, fibronectin, p300/CBP, NPM1, RPS2, DNMT3A, FUS, EWS, NCF1, NCF2, S100A3	Chromatin depolymerization, gene expression regulation, cell differentiation and apoptosis, innate immune response, NETosis process, tumor progression	rheumatoid arthritis [72,73], Alzheimer’s disease [74], multiple sclerosis [23], sepsis [24], inflammatory bowel disease [75], acute kidney injury [76,77], COVID-19 [25,78], cancer and thrombosis
PAD6	Ovary, early embryo, ovum, oocyte, testis, thymus	*α*-Tubulin	Cytoskeletal recombination in eggs and early embryos, preimplantation cleavage, early embryonic development, female fertility, contraceptive drug targets	Infertility [58,79]

**Table 2:** The potential role played by PAD4 in cancers and the associated mechanisms of PAD4 action.

Cancer Type	Biological Model	Regulatory Mechanisms	Biological Effects	References
Gastric carcinoma (GC)	1. Human gastric adenocarcinoma cell lines: MNK-45, SGC-7901, and AGS	PAD4 upregulates the expression of CXCR2, KRT14, TNF-α, and matrix metalloproteinases MMP2 and MMP9	Suppression of PADI4 decreases cell proliferation and invasion, results in S-phase arrest and fewer GC cells in the G2/M-phase. The combination of PADI4 short interfering RNA (siRNA) and 5-FU significantly inhibits GC cell proliferation.	[28,29,232,233,234]
	2. Human GC cell line: MGC80-3	PAD4 regulates IL-8 expression, upregulates Slug and vimentin expression, and downregulates E-cadherin expression	PAD4-induced epithelial-mesenchymal transition (EMT) and migration of GC cells are attenuated by inhibition of IL-8 expression.	
Ovarian carcinoma (OC)	1. Human OC cell lines: A2780 (p53 wild-type), and SKOV3 (p53-null)	Thirteen genes are related to cell proliferation, invasion, migration and apoptosis and their expression is differentially regulated	Silencing of PADI4 decreases the proliferation, invasion, and migration of p53-wt A2780 cells and increases the apoptosis rate, but only proliferation inhibition is observed with p53-null SKOV3 cells.	[28,29,235,236,237]
	2. Mouse OC cell line: ID8 Human OC cell line: ES2	PAD4 mediates the formation of NETs and promotes the premetastatic omental niche to facilitate the implantation of ovarian cancer cells	Inhibition of PAD4 blocks NET formation and reduces omental colonization of OC cells.	
Osteosarcoma (OS)	1. Human OS cell lines: U2OS and Saos-2 2. Xenograft nude mice model (injected with cells containing a *PADI4*-expressing lentivirus vector)	PAD4 activates the Wnt/*β*-catenin and MEK/ERK signaling pathways and promotes cell proliferation	Inhibition of PAD4 can reduce the proliferation and colony formation ability of OS cells.	[28,29,38,238,239]
		PAD4 downregulates the expression of E-cadherin in OS cells and induces the EMT	The expression of PAD4 is positively correlated with pulmonary metastasis in patients with OS. Inhibition of PAD4 expression interferes with the EMT of OS cells and inhibits their invasion and migration.	
		PAD4 and HDAC2 simultaneously bind to the p21 promoter and inhibit tumor cell apoptosis.	PAD4 inhibitor Cl-amidine or YW3-56 combined with the HDAC inhibitor SAHA exerts an additive effect in inhibiting the proliferation of U2OS cells.	
Nasopharyngeal carcinoma (NPC)	1. Human NPC cell lines: C666-1, 6-10B, and 5 2. Xenograft nude mice model (injected with C666-1 cells)	The LINC00324/miR-3164/PAD4 axis activates the PI3K/AKT pathway in NPC cells and promotes apoptosis and autophagy.	Silencing of PADI4 inhibits the proliferation and increases the apoptosis rates of NPC cells and induces autophagy.	[240,241,242]
		PAD4 downregulates the proapoptotic factors Bax, Bak, Bim and Bad; upregulates the anti-apoptotic factors Bcl-2, Bcl-xl and Mcl-1; decreases the proportion of cells in the G0/G1 phase; and promotes cell cycle progression.	PAD4 inhibitors GSK484 and YW3-56 restore the radiosensitivity of NPC cells; suppress cell proliferation, invasion, and migration; and significantly inhibit tumor growth in mice.	
Hepatic carcinoma	1. Human hepatocellular carcinoma (HCC) cell lines: SMMC-7721 and HepG2 2. Xenograft nude mice model (injected with SMMC-7721 cells)	PAD4 causes chemotherapy resistance of HCC cells by inducing autophagy.	Autophagy inhibitor effectively restores the sensitivity of HCC cells to chemotherapy in vitro and in vivo.	[28,29,186,243,244,245,246]
	3. The STAM model of nonalcoholic steatohepatitis (NASH)/HCC	Elevated free fatty acids stimulate NET formation in vitro	NET inhibition by DNase Ι or *PADI4* knockout (KO) changes the pattern of liver inflammation and reduces the progression of liver cancer in NASH patients.	
	4. Human HCC cell lines: HepG2 and MHCC97H 5. Mouse HCC cell line: Hepa1-6 C57BL/6 model mice (injected with Hepa1-6) with lipopolysaccharide (LPS)-induced NET formation	NETs activate the TLR4/9-COX2 signaling pathway in HCC cells and induce the inflammatory response, change the tumor inflammatory microenvironment, and promote tumorigenesis and metastasis cascades	The combination of DNase Ι with the anti-inflammatory drugs aspirin/hydroxychloroquine (HCQ) effectively attenuates HCC metastasis in mouse models.	
	6. Human HCC cell lines: HuH7 and MHCC97L 7. Mouse HCC cell line: Hepa1-6 Xenograft nude mice model (injected with HuH7 or 97L cells) 8. In a coinjection experiment (injected intravenously with HuH7 cells and human neutrophils)	HCC cell-derived IL-8 triggers NET formation mediated through the NADPH oxidase pathway	NET-associated cathepsin G promotes HCC metastasis in vitro and in vivo.	
Colon Cancer	1. Human colon cancer cell lines: SW480, SW620, DLD-1, HT29, HCT116, and LoVo 2. Mouse colon cancer cell line: MC38 3. SCID mouse models of hepatic metastases (injected with HT29, HCT116 or LoVo cells) 4. C57BL/6 mouse models of hepatic metastases (injected with MC38 cells)	PAD4 mediates citrullination of extracellular matrix (ECM) components; promotes colorectal cancer (CRC) cell adhesion; inhibits CRC cell mobility; increases the expression of the epithelial marker E-cadherin; and promotes the mesenchymal–epithelial transition (MET)	The PAD inhibitor BB-Cl-amidine or *PADI4* KO inhibits ECM citrullination, enhances mesenchymal cell marker expression, and inhibits liver metastasis growth. DNase Ι or PAD4 inhibitor YW4-03 inhibits NETs-mediated proliferation, adhesion, migration, and invasion of cancer cells. PAD4-KO tumors exhibit decreased mitochondrial density, mitochondrial DNA, ATP production and mitochondrial biogenesis proteins, causing decreased cell proliferation, an increased apoptosis rate and increased oxidative stress.	[86,188,189,247]
Lung carcinoma	Non-small-cell lung cancer (NSCLC) cell lines: 95D and A549 Xenograft nude mice model (injected with A549 cells)	Surgical stress promotes NET formation, alters the inflammatory microenvironment, and releases HMGB1 to activate TLR9-dependent pathways in cancer cells	The Au nanodrug delivery system YW3-56 targets tumors and when combined with photothermal therapy significantly inhibits tumor growth and lung metastasis in mice, showing good biosafety.	[28,29,122,248,249,250]
	NSCLC cell lines: HCC827 and H1650 Gefitinib-resistant cell lines: HCC827/G and H1650/G	NE released from NETs activates the TLR4-p38-PGC-1*α* axis in cancer cells to increase mitochondrial biosynthesis.	PAD4 expression is decreased in gefitinib-resistant cell lines, and the upregulation of PAD4 expression reverses the resistance of NSCLC cell lines to gefitinib by inhibiting Elk-1 expression and the EMT.	
Breast carcinoma	1. Human breast carcinoma cell lines: MCF-7 and T47D	PAD4 citrullinates the N-terminal domain of GSK3*β*, promoting nuclear GSK3*β* accumulation, inhibiting Smad stability and TGF-*β* signaling, thereby ensuring maintenance of epithelial phenotypes	Silencing of *PADI4* in MCF-7 cells induces the EMT and greater invasiveness of tumors, as shown in xenograft experiments.	[28,29,89,90,251,252]
	2. Human breast carcinoma cell lines: MCF-7 and MCF-7/ADR	PAD4 increases the nuclear levels of GSK3β and p53, which activate proapoptotic gene expression and downregulate MDR1 expression	Restoring PAD4 expression in MCF-7/ADR cells reverses drug resistance by inducing apoptosis.	
	3. Human breast carcinoma cell lines: MCF10CA1h, MCF10CA1a, MCF-7E, and MDA-MB-231 LM2 4. Female athymic NCI nu/nu mouse model (mammary fat pad injection with MCF-7 cells)	PAD4 downregulates the expression of stemness master transcription factors NANOG and OCT4 by reducing the transcriptional activation mediated through H3R17me2a marked loci	Endogenous PAD4 activity limits the amount of cancer stem cells (CSCs) residing in multiple breast cancer models in vitro and in vivo.	
	5. Mouse triple-negative breast cancer (TNBC) cell lines: 4T1 and 67NR 6. Allograft female BALB/c mice model (with 4T1 cells injected into mammary fat pad) 7. BALB/c mouse model of pulmonary metastasis (with 4T1 cells injected intravenously)	PAD4 mediates histone hypercitrullination, and cancer extracellular chromatin network (CECN) release from 4T1 cells	*PADI4* KO in 4T1 cells inhibits tumor growth and lung metastasis in mice. Degradation of extracellular DNA by DNase Ι inhibits pulmonary metastasis of *PADI4*-induced 4T1 cells.	[222,253,254]
	8. Human TNBC cell lines: MDA-MB-231 and BT-549 9. Xenograft nude mouse model (injected with MDA-MB-231 cells)	GSK484 facilitates TNBC cell apoptosis, offsets irradiation (IR)-induced upregulation of ATG5 and ATG7 expression and downregulation of p62 expression, while enhancing DNA damage.	GSK484 pretreatment enhances IR-induced inhibition of cell proliferation, migration, and invasion. The combination of IR and GSK484 significantly inhibits tumor growth and metastasis.	
	10. Human TNBC cell line: MDA-MB-231 11. Xenograft SCID mouse model (MDA-MB-231 cells injected with into the mammary fat pad)	Hypoxia-inducible factors (HIFs) recruit PAD4 to the hypoxia response elements (HREs) of HIF target genes under hypoxic conditions and stimulate transcriptional activation	PAD4 promotes breast cancer angiogenesis and tumor growth.	
	12. Dormancy model (inflammation induced through intranasal administration of LPS): 13. BALB/c mice (injected intravenously with mCherry-luciferase-D2.0R cells), nude mice (injected intravenously with MCF-7 cells) 14. BALB/c mouse model of pulmonary metastasis (injected intravenously with 4T1 or MDA-MB-231 cells)	NETs release NE and MMP9 and reshape laminin, activating the integrin *α*3*β*1 pathway and FAK/ERK/MLCK/YAP signaling to awaken dormant cancer cells.	Sustained inflammation induces NET formation and promotes the awakening of dormant cancer cells. The PAD4 inhibitor GSK484 and DNase I prevent the reawakening of dormant D2.0R and MCF-7 cancer cells. Ozone exposure increases lung neutrophil inflammation in mice, promoting NET release and cancer cells colonization of the lung.	[200,255]
Leukemia	1. Acute promyelocytic leukemia (APL) cells: HL-60 (PML-RAR*α-* negative) and NB4 (PML-RAR*α-* positive)	PAD4 inhibition in APL inhibits cell differentiation mediated through the functional PAD4/SOX4/PU.1 axis	All-trans-retinoic acid (ATRA) treatment restores PAD4 expression to promote its nuclear translocation in APL cells, and it promotes the differentiation of leukemia cells by regulating PU.1 expression.	[109,113,256,257]
	2. Bone marrow cells isolated from PAD4-KO mice and wt C57BL/6 mice	PAD4 regulates the proliferation of MPP cells by binding LEF1 and HDAC1 to control c-myc expression.	Compared with cells from wt mice, LSK cells, especially MPP cells, proliferate abnormally in PAD4-KO mice.	
	3. Human cell lines: HL-60, Jurkat T, and Jurkat-Tet-On	PAD4 overexpression increases the accumulation of p53 in hematopoietic cells, stimulates the transcription of P21 and Bax, and induces apoptosis and cycle arrest.	PAD4 reduces HL-60 and Jurkat cellular activity in a dose- and time-dependent manner	

**Table 3:** An overview of PAD4 inhibitors.

Type	Inhibitor	Structure	IC_50_	Reference
Reversible inhibitors	Taxol	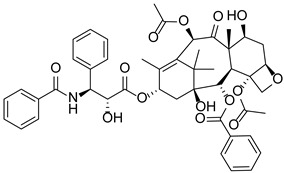	IC50 = ≈5 mM	[94,290]
	Bz-ADMA	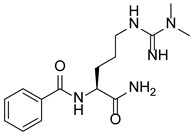	IC50 = 0.4 mM	[31,291]
	Streptomycin	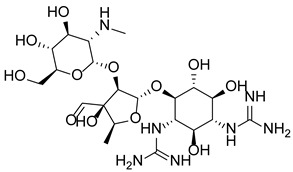	IC50 = 1.8 mM	[31,292]
	Minocycline	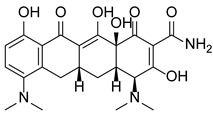	IC50 = 0.62 mM	[31,40,292]
	Chlortetracycline	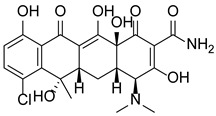	IC50 = 100 μM	[31,40,292]
	GSK199	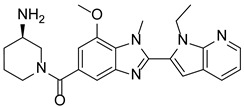	IC50 (0 mM Ca) = 200 nM; IC50 (2 mM Ca) = 1.0 μM	[31,40,168]
	GSK484	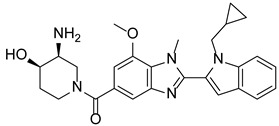	IC50 (0 mM Ca) = 50 nM; IC50 (2 mM Ca) = 250 nM	[31,40,168]
	Inh-Dap	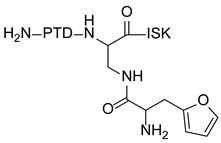	IC50 = 243.2 ± 2.4 μM	[293]
	SC97362	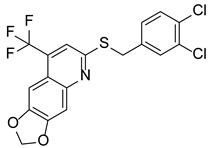	IC50 = 1.88 ± 0.26 μM	[294]
Irreversible inhibitors	NSC95397	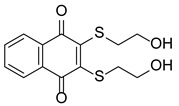	kinact/KI (M^-1^·min^−1^) PAD1: 175 PAD2: 1600 PAD3: 9150 PAD4: 4530	[40,295]
	Streptonigrin	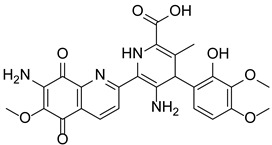	kinact/KI (M^−1^·min^−1^) PAD1: 3700 PAD2: 12,000 PAD3: 3500 PAD4: 440,000	[40,296]
	Cl-amidine	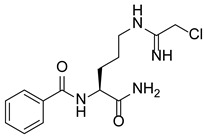	IC50 = 6 μM kinact/KI (M^−1^ min^−1^) PAD1: 37,000 PAD2: 1200 PAD3: 2000 PAD4: 13,000	[31,40,297,298]
	F-amidine	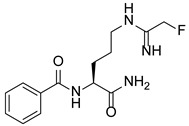	IC50 = 22 μM kinact/KI (M^−1^·min^−1^) PAD1: 2800 PAD2: 380 PAD3: 170 PAD4: 3000	[31,40,297,298]
	*o*-Cl-amidine	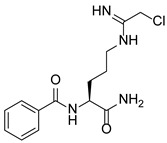	IC50 = 2.2 μM kinact/KI (M^−1^·min^−1^) PAD1: 106,400 PAD2: 14,100 PAD3: 10,345 PAD4: 38,000	[31,40,298]
	*o*-F-amidine	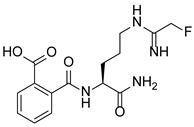	IC50 = 1.9 μM kinact/KI (M^−1^·min^−1^) PAD1: 180,900 PAD2: 7500 PAD3: 6700 PAD4: 32,500	[31,40,298]
	BB-Cl-amidine	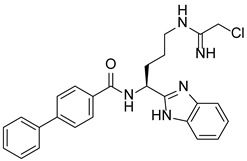	kinact/KI (M^−1^·min^−1^) PAD1: 16,100 PAD2: 4100 PAD3: 6800 PAD4: 13,300	[31,40,299]
	BB-F-amidine	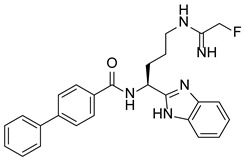	kinact/KI (M^−1^·min^−1^) PAD1: 900 PAD2: 1200 PAD3: 3400 PAD4: 3750	[31,40,299]
	TDFA	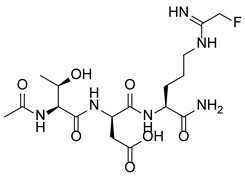	IC50 = 2.3 μM kinact/KI (M^−1^·min^−1^) PAD1: 1700 PAD2: 500 PAD3: 400 PAD4: 26,000	[31,40,300]
	TDCA	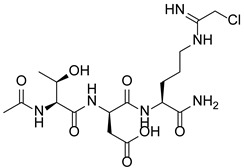	IC50 = 3.4 μM kinact/KI (M^−1^·min^−1^) PAD1: 21,000 PAD2: 300 PAD3: 920 PAD4: 24,000	[31,40,300]
	YW3-56	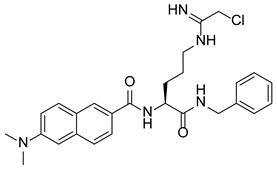	IC50 = 1–5 μM	[111]
	ZD-E-1M	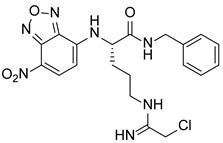	IC50 = 2.39 μM	[301]

## Abbreviations

CAT	Cancer-Associated Thrombosis
PAD4	Peptidyl arginine deiminase 4
VTE	Venous thromboembolism
ER	Estrogen receptor
PTM	Post-translational modification
NLS	Nuclear localization sequence
CRPC	Castration-resistant prostate cancer
HDAC1	Histone deacetylase 1
HDAC2	Histone deacetylase 2
ES	ES OCT4-GIP
iPS	Induced pluripotent stem cells
ATRA	All-trans retinoic acid
mTORC1	Mammalian target of rapamycin complex 1
CK	Cytokeratin
AT	Antithrombin
EMT	Epithelial–mesenchymal transition
GSK3*β*	Glycogen synthase kinase 3*β*
ETS	Erythroblast transformation-specific
EGF	Epidermal growth factor
NSCLC	Non-small cell lung cancer
CRC	Colorectal cancer
ECM	Extracellular matrix
NPM1	Nucleophosmin
RPS2	Ribosomal protein S2
ROS	Reactive oxygen species
NETs	Neutrophil extracellular traps
NE	Neutrophil elastase
MPO	Myeloperoxidase
MMP9	Matrix metalloproteinase 9
CG	Cathepsin G
PMA	Phorbol myristate acetate
Abs	Antibodies
PKC	Protein kinase C
TLRs	Toll-like receptors
GM-CSF	Granulocyte-macrophage colony stimulating factor
LPS	Lipopolysaccharide
C5a	Complement factor 5a
H3Cit	Citrullinated histone H3
G-CSF	Granulocyte colony-stimulating factor
HMGB1	High-mobility group box 1
CTLs	Cytotoxic T lymphocytes
NKs	Natural killer cells
cfDNA	Cell-free DNA
TFPI	Tissue factor pathway inhibitor
PSGL-1	p-selectin glycoprotein ligand-1
DAMPs	Damage-associated molecular patterns
GC	Gastric carcinoma
OC	Ovarian carcinoma
OS	Osteosarcoma
NPC	Nasopharyngeal carcinoma
PHC	Primary hepatic carcinoma
HCC	Hepatocellular carcinoma
ICC	Intrahepatic cholangiocarcinoma
EVs	Extracellular vesicles
MET	Mesenchymal-epithelial transition
CSC	Cancer stem cell
TNBC	Triple-negative breast cancer
CECNs	Cancer extracellular chromatin networks
HIFs	Hypoxia-inducible factors
HREs	Hypoxia response elements
APL	Acute promyelocytic leukemia
HIT	Heparin-induced thrombocytopenia
MDSCs	Myeloid suppressor cells
